# An alternate mechanism for the emergence of localized oscillatory patterns in networked reaction–diffusion systems

**DOI:** 10.1093/nsr/nwag523

**Published:** 2026-09-02

**Authors:** Zhi-Chao Xue, Xiaofeng Luo, Li Li, Yongli Song, Stefano Boccaletti, Charo I del Genio, Yicheng Zhang, Gui-Quan Sun

**Affiliations:** Complex Systems Research Center, Shanxi University, Taiyuan 030006, China; School of Mathematics, North University of China, Taiyuan 030051, China; Sino-Europe Complex Science Center, North University of China, Taiyuan 030051, China; School of Computer and Information Technology, Shanxi University, Taiyuan 030006, China; School of Mathematics, Hangzhou Normal University, Hangzhou 311121, China; Sino-Europe Complex Science Center, North University of China, Taiyuan 030051, China; Institute of Interdisciplinary Intelligent Science, Ningbo University of Technology, Ningbo 315211, China; CNR-Institute of Complex Systems, Sesto Fiorentino I-50019, Italy; Institute of Interdisciplinary Intelligent Science, Ningbo University of Technology, Ningbo 315211, China; Institute of Biophysics and Biomedical Engineering, Bulgarian Academy of Sciences, Sofia 1113, Bulgaria; Sino-Europe Complex Science Center, North University of China, Taiyuan 030051, China; Physics Department, University of Fribourg, Fribourg 1700, Switzerland; Complex Systems Research Center, Shanxi University, Taiyuan 030006, China; School of Mathematics, North University of China, Taiyuan 030051, China; Sino-Europe Complex Science Center, North University of China, Taiyuan 030051, China

**Keywords:** complex network, localized oscillatory patterns, networked system, subcritical branch

## Abstract

Localized oscillatory patterns, confined to finite spatial domains or subsets of nodes in a network, have been observed in cortical neurons, metacommunity ecosystems, and chemical experiments.Previous studies have shown that wave bifurcations (oscillatory Turing instabilities) in undirected networked reaction–diffusion systems can induce such localized oscillations. Here, we propose an alternative mechanism for their emergence, namely Hopf-type instabilities acting on a subcritical Turing branch. Unlike wave bifurcations, this mechanism does not require interactions amongst at least three components. We further demonstrate its robustness in both the Brusselator and FitzHugh–Nagumo models in undirected Barabási–Albert, Erd˝os–Rényi, and Watts–Strogatz networks. This work extends the universality of such patterns in networked systems and provides a theoretical foundation for the control of collective dynamical phenomena in complex systems.

## Teaser

This study reveals how complex networks sustain intricate spatial organization through surprisingly simple dynamics, with localized oscillations emerging from a minimal Turing-Hopf mechanism without multi-component interactions.

## INTRODUCTION

The formation of spatiotemporal patterns is a central problem in the study of complex systems and nonlinear dynamics [1–3]. The idea of using reaction–diffusion systems to explain pattern formation was first introduced by Turing in 1952, providing a prototypical framework for diffusion-driven instabilities [4]. Although the theory was initially developed from a purely theoretical perspective, its validity was not experimentally confirmed until the early 1990s, when Castets *et al.* first observed stationary chemical patterns [5], followed by the experimental realization of Turing patterns in the chlorite–iodide–malonic acid reaction system [6,7]. These pioneering experiments established diffusion-driven instability as a general mechanism for spontaneous pattern formation and stimulated extensive research across disciplines, such as ecology [8] and biology [9]. Within this classical reaction–diffusion framework, Turing instabilities produce stationary spatial structures, whereas Hopf instabilities give rise to temporal oscillations. Together, they underpin a broad spectrum of spatiotemporal behaviors and provide the theoretical foundation for understanding self-organization in complex systems.

In many real-world systems, however, spatial organization is inherently discrete and heterogeneous rather than continuous [10]. Representative examples include metapopulation ecosystems, where habitats are represented as discrete patches connected by migration pathways [11–15], and epidemic spreading across transportation or mobility networks, where long-range movement occurs through heterogeneous connections [16–18]. These systems motivate a network-based representation of space, in which nodes correspond to spatial units and links describe migration or mobility pathways. From this perspective, networked reaction–diffusion models provide a natural complement to classical continuous reaction–diffusion systems, extending the study of pattern formation to spatially discrete domains while retaining the same reaction–diffusion principles.

Over the past decade, advances in network science have revealed that network topology plays a fundamental role in shaping dynamical processes across diverse complex systems. In biological and ecological networks, transient amplification has been shown to provide a more informative predictor of extinction risk than asymptotic stability [19]. More broadly, structural heterogeneity, clustering, and temporal interactions have been identified as key determinants of collective behavior, evolutionary dynamics, and network controllability [20–25]. Recent studies in network neurodynamics further demonstrate that the interplay between network architecture and nonlinear dynamics governs transitions among multiple neural states through attractor-based mechanisms [26,27]. These findings underline the distinct advantages of complex networks in capturing structural heterogeneity and temporal variability. Consequently, reaction–diffusion systems have been extended to complex network topologies [10,28–34], highlighting the crucial role of network structure in pattern formation.

Notably, globally coherent states are not the only instances of spatiotemporal patterns that can emerge in complex systems. In fact, localized oscillatory patterns, in which temporal oscillations are confined to a subset of nodes or regions, are a somewhat common occurrence, having been observed in a large number of situations, including granular media, physical reaction–diffusion systems, neuronal networks, and metapopulation systems [35–46].

In continuous media, localized patterns with an oscillatory background have been widely studied, and are generally interpreted as the result of Turing–Hopf interactions. Nonetheless, only a few works have considered the emergence of patterns involving only a subset of nodes in networked reaction–diffusion systems. The mechanism suggested to cause the phenomenon is that of wave bifurcations (oscillatory Turing instabilities) [47], which, however, requires at least three interacting components, similarly to what happens in continuous systems [4,48,49]. This fundamental requirement restricts the applicability of the oscillatory Turing framework to systems with three or more dynamical variables, leaving the origin of localized oscillatory patterns in the broad class of two-component reaction–diffusion systems largely unexplored.

However, in many real-world systems, the number of dynamical components is intrinsically constrained by the underlying biological or physical mechanisms. For example, in cortical neuroscience, the interactions between excitatory and inhibitory neuronal populations constitute the minimal framework for describing large-scale brain dynamics [50]. Likewise, in epidemiology, the spread of infectious diseases without permanent immunity is naturally captured by the two-component Susceptible–Infected–Susceptible model [51]. In such systems, introducing additional dynamical variables is often neither biologically meaningful nor physically justified. These considerations motivate a fundamental question: can localized oscillatory patterns arise in networked reaction–diffusion systems with only two interacting components? Addressing this question would extend the applicability of localized oscillation theory while preserving the mechanistic consistency and interpretability of low-dimensional models.

Here, we propose an alternative mechanism for the occurrence of localized oscillatory patterns in two-dimensional reaction–diffusion systems on undirected networks. Specifically, we show that such states can be generated by Hopf-type instabilities acting on a subcritical Turing branch. This mechanism overcomes the component-number limitation inherent to traditional oscillatory Turing instability theory. Moreover, by applying our approach to different systems across three types of networks, we show the consistent emergence of these localized oscillatory patterns, highlighting their universality.

## RESULTS

### The model

Consider a general reaction–diffusion system with two species in an undirected and unweighted network of *N* nodes, whose evolution is given by


(1) \begin{eqnarray*}
\frac{\partial }{\partial \mathrm{t}}\mathbf {U}_i(t) = \mathbf {F}(\mathbf {U}_i) + \mathbf {D} \sum _{j=1}^{N} L_{ij} \mathbf {U}_j\:.
\end{eqnarray*}

Here, $\mathbf {U}_i$ is the two-dimensional vector of species concentrations at node *i*, *i=1,… ,N* and $t\in [0,\mathrm{T}]$, the vector function $\mathbf {F}$ encodes the local reaction kinetics, $\mathbf {D}$ is the diagonal diffusion matrix, whose elements are the diffusion coefficients of the corresponding species. $L_{ij}$ is the *(i,j)* element of the Laplacian matrix $\mathbf {L}$, defined as $\mathbf {L} = \mathbf {A}-\mathbf {K}$, where $\mathbf {A}=(A_{ij})$ is the adjacency matrix of the network, describing the connectivity between nodes, with


(2) \begin{eqnarray*}
A_{ij}= \left\lbrace \begin{array}{@{}l@{\quad }l@{}}1, & \text{if nodes } i \text{ and } j \text{ are connected},\\
0, & \mathrm{otherwise}, \end{array}\right.
\end{eqnarray*}

and $\mathbf {K}=\mathrm{diag}(k_1,\dots ,k_N)$ is the diagonal degree matrix characterizing the number of connections of each node, whose diagonal entries are given by


(3) \begin{eqnarray*}
k_i=\sum _{j=1}^{N} A_{ij}.
\end{eqnarray*}

Equivalently,


(4) \begin{eqnarray*}
L_{ij}=A_{ij}-k_i\delta _{ij},
\end{eqnarray*}

where $\delta _{ij}$ is the Kronecker delta. Note that, under this definition, the network Laplacian $\mathbf {L}$ is negative semi-definite.

Now, assume that the system admits a homogeneous steady state $\mathbf {U}^{*}$. If the state $\mathbf {U}_i(t)$ is obtained by adding a small perturbation $\!\!\tilde{\mathbf {\,\,\,U}}_i$ to $\mathbf {U}^{*}$, then a linearization of the system around the steady state produces


(5) \begin{eqnarray*}
\frac{\partial }{\partial \mathrm{t}}\!\!\tilde{\mathbf {\,\,\,U}}_i = \left. J\mathbf {F}\right|_{\mathbf {U}^{*}} \!\!\tilde{\mathbf {\,\,\,U}}_i + \mathbf {D} \sum _{j=1}^{N} L_{ij} \!\!\tilde{\mathbf {\,\,\,U}}_j\:,
\end{eqnarray*}

where *J* is the Jacobian operator.

The Laplacian eigenvectors form an orthonormal basis of $\mathbb {R}^N$. Let


(6) \begin{eqnarray*}
\mathbf {V} = (\boldsymbol{\phi }^{(1)},\dots ,\boldsymbol{\phi }^{(N)})
\end{eqnarray*}

be the orthogonal matrix whose columns are these eigenvectors, which satisfy the standard Laplacian eigenvalue problem:


(7) \begin{eqnarray*}
\mathbf {L} \boldsymbol{\phi }^{(\alpha )} = \Lambda _\alpha \boldsymbol{\phi }^{(\alpha )}, \quad \alpha = 1, \dots , N,
\end{eqnarray*}

where $\Lambda _\alpha$ is the *α*th eigenvalue associated with the eigenvector $\boldsymbol{\phi }^{(\alpha )}$. In component form, this eigenvalue problem can be expressed as $\sum _{j=1}^N L_{ij}\phi _j^{(\alpha )} = \Lambda _\alpha \phi _i^{(\alpha )}$. Following [28], we expand the perturbation $\!\!\tilde{\mathbf {\,\,\,U}}_i$ on this orthonormal basis. Specifically, the perturbation at the *i*th node is expressed as


(8) \begin{eqnarray*}
\!\!\tilde{\mathbf {\,\,\,U}}_i(t) = \sum _{\alpha =1}^N \mathbf {c}_{\alpha } \mathrm{e}^{\lambda _{\alpha } t} \phi _i^{(\alpha )}\:,
\end{eqnarray*}

where *α* denotes the mode index. Here, $\lambda _\alpha$ determines the temporal growth or decay rate of mode *α*, and $\phi _i^{(\alpha )}$ represents the *i*th component of the *α*th Laplacian eigenvector $\boldsymbol{\phi }^{(\alpha )}$. Note that Equation (8) requires the Laplacian matrix to be diagonalizable [29]. Since we focus on undirected random networks, the Laplacian matrix $\mathbf {L}$ is real and symmetric, and therefore admits a complete orthonormal basis represented by an orthogonal matrix $\mathbf {V}$. It is worth noting that, in random networks, the absence of structural symmetries typically prevents exact spectral degeneracies [52]. The repeated Laplacian eigenvalues are nongeneric in the networks considered here. Therefore, in the following bifurcation analysis and center manifold reduction, we focus on the generic case where the critical Laplacian eigenvalue is simple.

Substituting expansion (8) into Equation (5) yields, for each mode *α*, the problem


(9) \begin{eqnarray*}
\lambda _{\alpha } \mathbf {I} = \left. J\mathbf {F}\right|_{\mathbf {U}^{*}} + \Lambda _{\alpha } \mathbf {D}\:,
\end{eqnarray*}

where $\mathbf {I}$ is the two-dimensional identity matrix and $\Lambda _{\alpha }$ is the *α*th eigenvalue of the Laplacian. Then, the linear stability of the homogeneous steady state is determined by the eigenvalue with the largest real part, so that the state is stable if $\displaystyle \max _{\alpha }\left\lbrace \boldsymbol\Re \lambda _{\alpha }\right\rbrace < 0$, and unstable if $\displaystyle \max _{\alpha }\left\lbrace \boldsymbol\Re \lambda _{\alpha }\right\rbrace =0$.

We can thus identify bifurcation conditions straightforwardly. First, a Hopf bifurcation arises when a pair of complex conjugate eigenvalues of the Jacobian matrix crosses the imaginary axis at the null spatial mode. However, in networked systems, the role of the spatial modes is played by the Laplacian eigenvalues $\Lambda _{\alpha }$. Since $\mathbf {L}$ always has at least one null eigenvalue $\Lambda _1$, the condition for the Hopf bifurcation becomes


(10) \begin{eqnarray*}
\boldsymbol\Re \lambda _1 = 0,\quad \boldsymbol\Im \lambda _1\ne 0,
\end{eqnarray*}

where $\lambda _1$ is the value of $\lambda _{\alpha }$ that solves Equation (9) for $\Lambda _\alpha =\Lambda _1=0$, that is, $\lambda _1$ is one of the eigenvalues of the Jacobian of $\mathbf {F}$ computed on the homogeneous steady state.

In contrast, a Turing bifurcation arises from the destabilization of a non-homogeneous mode associated with a Laplacian eigenvalue $\Lambda _T< 0$. The instability threshold is reached when the corresponding $\lambda _T$ satisfies


(11) \begin{eqnarray*}
\boldsymbol\Re \lambda _T=0,\quad \boldsymbol\Im \lambda _T=0,
\end{eqnarray*}

so that a purely real eigenvalue crosses the origin.

If both instability conditions are satisfied, the steady-state (Turing) branch is influenced by a Hopf-type instability, allowing stationary patterns to acquire oscillatory features.

### The Brusselator model

Here, we focus on the Brusselator model [53,54], which serves as a paradigmatic example for the emergence of Hopf and Turing instabilities in reaction–diffusion systems.

In this model, each node has a state vector $\mathbf {U}_i=(u_i, v_i)^{\mathrm{T}}$, *i=1,… ,N*, where $u_i$ and $v_i$ denote the concentrations of an activator and an inhibitor species, respectively. The local reactions at each node are described by


(12) \begin{eqnarray*}
\mathbf {F}(\mathbf {U}_i) = \left(\begin{array}{c}
{p - \left(r+1\right)u_i + u_i^2 v_i}\\
{r u_i - u_i^2 v_i}\end{array}\right),
\end{eqnarray*}

where *p> 0* and *r> 0* are control parameters. Also, we write the diffusion matrix as


(13) \begin{eqnarray*}
\mathbf {D} = \left({\begin{array}{cc}
\varepsilon & 0 \\
0 & \varepsilon \sigma \end{array}}\right),
\end{eqnarray*}

where *ɛ > 0* sets the overall diffusion strength, and *σ > 0* denotes the ratio between inhibitor and activator diffusivities.

This system admits a spatially homogeneous equilibrium state


(14) \begin{eqnarray*}
\mathbf {U}^{*} = \left(p, \frac{r}{p}\right),
\end{eqnarray*}

so that


(15) \begin{eqnarray*}
\left. J\mathbf {F}\right|_{\mathbf {U}^{*}} =
\left({\begin{array}{cc}
r-1 & p^2\\
-\, r & -p^2 \end{array}}\right).
\end{eqnarray*}

Since the system is two-dimensional, Equation (9) can be rewritten as


(16) \begin{eqnarray*}
\lambda _{\alpha }^2 - \lambda _{\alpha } T + \Delta = 0,
\end{eqnarray*}

where we have put


(17) \begin{eqnarray*}
T = \mathrm{Tr}\left(\left. J\mathbf {F}\right|_{\mathbf {U}^{*}} + \Lambda _{\alpha }\mathbf {D}\right)
\end{eqnarray*}

and


(18) \begin{eqnarray*}
\Delta = \left\Vert \left. J\mathbf {F}\right|_{\mathbf {U}^{*}} + \Lambda _{\alpha }\mathbf {D}\right\Vert .
\end{eqnarray*}

This allows us to determine the phase portrait of the system from the relation between *T* and *Δ*. In particular, since a Hopf bifurcation requires a pair of complex conjugate eigenvalues to cross the imaginary axis, we need $T^2-4\Delta < 0$. At the same time, we know that this needs to happen for the zero spatial mode. Thus, a Hopf bifurcation requires simultaneously *T=0* and *Δ > 0*. Moreover, it corresponds to the homogeneous mode associated with the null Laplacian eigenvalue $\Lambda _{\alpha }=\Lambda _1=0$. Therefore, we obtain $T=r-1-p^2=0$, which yields


(19) \begin{eqnarray*}
r = p^2+1.
\end{eqnarray*}

In turn, this results in $\Delta =-p^4+p^2\left(p^2+1\right)=p^2$, which is always positive, since *p> 0*. Thus, the condition *Δ > 0* is automatically satisfied.

Conversely, a Turing bifurcation arises from a real eigenvalue crossing the origin, which requires *Δ =0*. For each non-zero Laplacian eigenvalue ($\Lambda _{\alpha }\ne 0$), explicitly solving the resulting condition for *Δ* yields the relationship between the control parameters:


(20) \begin{eqnarray*}
r = \frac{\left(\varepsilon \Lambda _T-1\right)\left(p^2 -\varepsilon \sigma \Lambda _T\right)}{\varepsilon \sigma \Lambda _T},
\end{eqnarray*}

which determines the onset of stationary spatial patterns.

### Reduced dynamics

To determine the initial bifurcation direction and the local stability of the steady-state branch, we use normal form theory [55] to derive a reduced equation for the evolution of the system (Material S1), which is given by


(21) \begin{eqnarray*}
\frac{\mathrm{d}}{\mathrm{dt}}z = C_{11}\mu z + C_{20}z^2 + C_{21}\mu z^2 + C_{30}z^3,
\end{eqnarray*}

where *μ* is a reduced control parameter and $C_{11}$, $C_{20}$, $C_{21}$, and $C_{30}$ are coefficients whose explicit expressions are given in the Material S1.

Note that these coefficients represent the projection of the nonlinearities of the full system onto the center manifold. In particular, the linear coefficient $C_{11}$ determines the rate of exponential growth of perturbations along the critical eigenmode. The quadratic coefficient $C_{20}$ arises from symmetry-breaking nonlinearities in the reaction kinetics, introducing a directional bias in the initial growth phase. The cubic coefficient $C_{30}$ determines the type of bifurcation by its sign, with a negative value yielding a supercritical bifurcation, and a positive one giving rise to a subcritical bifurcation in which an unstable finite-amplitude pattern branch folds back, creating the hysteresis loop and the snaking structure that are necessary for the existence of localized states [56–58]. Finally, the mixed term $C_{21}$ measures the importance of the control parameter for the quadratic nonlinearity.

Thus, following the analysis of the stability of the reduced system detailed in Material S1, one can employ numerical continuation techniques (Material S2) to identify the region of the parameters where a subcritical Turing branch is subject to a Hopf instability, thereby determining where localized oscillatory patterns may arise.

### Stability analysis

To test our method on the Brusselator, we create a Barabási–Albert (BA) preferential-attachment network [59] with 30 nodes starting from 5 initial fully connected nodes. Based on the instability conditions given in Material S1, we determine the Hopf and Turing bifurcation points and assess the subcriticality of $r_T$ from the value of $C_{30}$.

Then, we construct the bifurcation diagram on the *(r,p)* parameter plane, as shown in Fig. 1a. The diagram is partitioned by a Hopf bifurcation line and a Turing bifurcation line into four distinct regions, namely a stable region, one of pure Turing instability, one of pure Hopf instability and one of simultaneous Turing and Hopf instabilities. To identify the critical modes, we study the real and imaginary parts of the growth rate *λ* for each of the Laplacian eigenvalues in each of the regions, as reported in the Fig. 1b–e, focusing on the region where Hopf and Turing instabilities co-occur.

**Figure 1.**
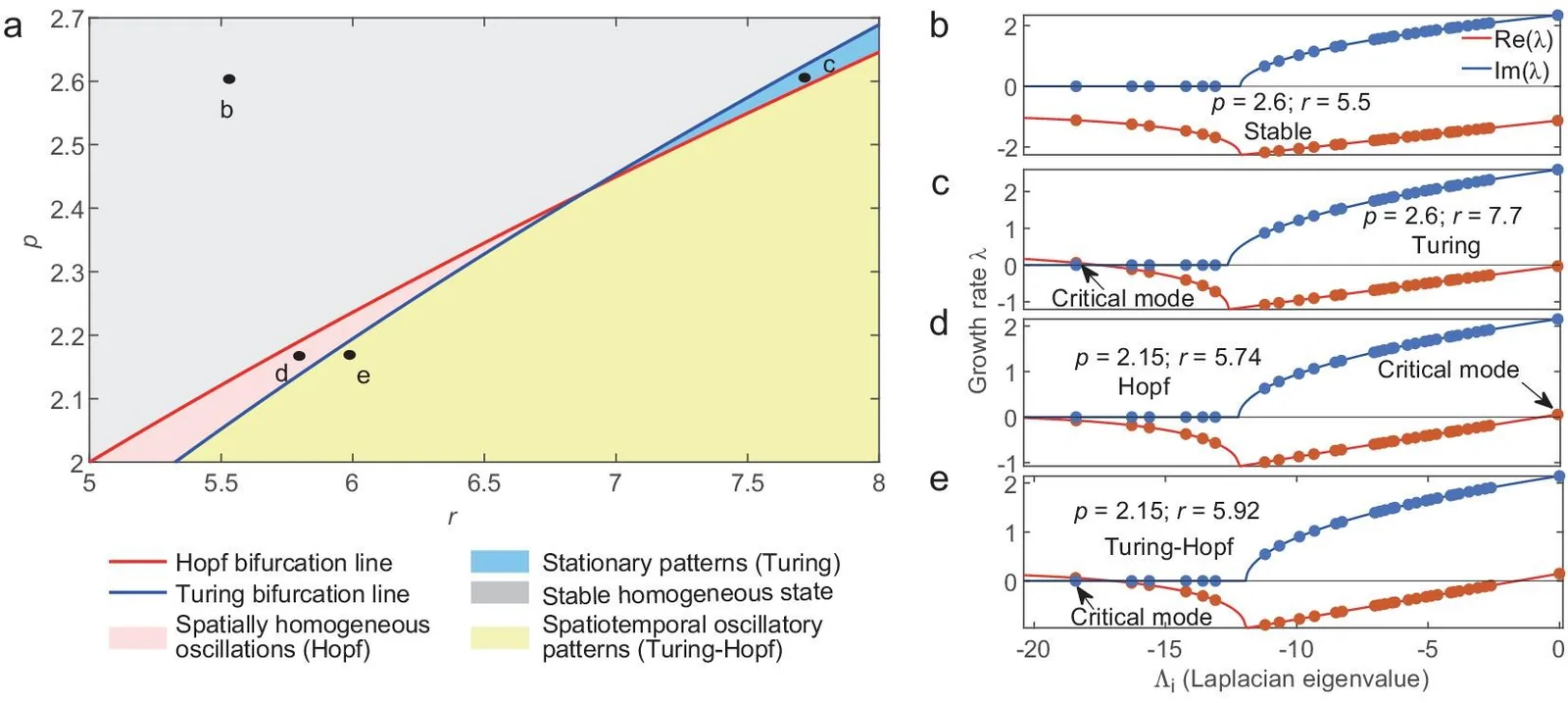
Stability analysis of the Brusselator model. (a) The bifurcation diagram in the *(r,p)* plane shows the existence of four regions, namely a stable one (gray, b; stable homogeneous state), a Turing-unstable one (blue, c; stationary patterns), a Hopf-unstable one (pink, d; spatially homogeneous oscillations), and one where Turing and Hopf instabilities coexist (yellow, e; spatiotemporal oscillatory patterns). The red curve denotes the Hopf bifurcation line, whereas the blue curve denotes the Turing bifurcation line. (b–e) The real and imaginary parts of the growth rate *λ* of each mode as functions of the corresponding Laplacian eigenvalues allow us to identify the critical modes for the corresponding points marked on the phase diagram in panel (a). The parameters used were *p=2.6* and *r=5.5* in (b), *p=2.6* and *r=7.7* in (c), *p=2.15* and *r=5.75* in (d), and *p=2.15* and *r=5.92* in (e). The other parameters were *ɛ =0.055* and *σ =2.4* in all cases.

To explore the origin of localized oscillatory patterns from the perspective of the branch structure of the solution, we first fix the parameters to *ɛ = 0.055*, *σ = 2.4* and *p = 2.15*, corresponding to the point in region (e) marked in Fig. 1a. Then, using the procedure described in Material S1, we compute the coefficients in Equation (21), obtaining


(22) \begin{eqnarray*}
\frac{\mathrm{d}}{\mathrm{dt}}z &=& 0.75351\mu z + 0.58098 z^2\\
&&+\, 0.029259\mu z^2 + 0.19007 z^3,
\end{eqnarray*}

which indicates that the bifurcation is subcritical.

### Global branch structure and pattern emergence

To characterize the emerging spatiotemporal patterns, we introduce two quantitative measures describing their spatial amplitude and oscillatory activity.

Following Ref. [28], the pattern amplitude at the final time T is defined as


(23) \begin{eqnarray*}
A = \sqrt{\sum _{i=1}^N [\left(u_i(\mathrm{T}) - u^{*}\right)^2 + \left(v_i(\mathrm{T}) - v^{*}\right)^2]},
\end{eqnarray*}

where *i=1,… ,N*, and $u^{*}$ and $v^{*}$ are the homogeneous steady state values of the two components.

To quantify oscillatory activity, we define the amplitude of single node *i* as


(24) \begin{eqnarray*}
A_i=\max _{t\in [t_1,t_2]}u_i(t)-\min _{t\in [t_1,t_2]}u_i(t),
\end{eqnarray*}

and $[t_1,t_2]$ denotes one oscillation period. Node *i* is classified as oscillatory if


(25) \begin{eqnarray*}
A_i> \eta \max _{1\le j\le N}A_j,
\end{eqnarray*}

where *η* is a small relative threshold used to distinguish genuine oscillations from weak residual fluctuations caused by numerical noise or finite-time transients. Throughout this work, we fix *η =0.05*.

Let $N_{\mathrm{osc}}$ denote the number of oscillatory nodes, and define the oscillation index:


(26) \begin{eqnarray*}
R=\frac{N_{\mathrm{osc}}}{N}.
\end{eqnarray*}

This index measures the fraction of oscillatory nodes in the network. Accordingly, (*R=0*) corresponds to non-oscillatory states, including homogeneous steady states and stationary spatial patterns; (*0< R< 1*) characterizes localized oscillatory patterns, in which oscillations are confined to a subset of nodes; and (*R=1*) corresponds to globally oscillatory states where all nodes exhibit sustained oscillations.

Using these two indices to characterize the solution branches, we next investigate their global organization by numerical continuation with AUTO-07P [60]. The resulting solution branch structure is illustrated in Fig. 2a, where the black line represents the homogeneous steady state. As *r* increases, the first bifurcation point detected (HB1) is a Hopf bifurcation at $r_H \approx 5.6225$, consistent with the theoretical prediction. At this bifurcation point, a new branch emerges, corresponding to temporally periodic oscillatory patterns, which are illustrated in Fig. 2b, characterized by an oscillation index of *R=1* in Equation (26). Note that, in Fig. 2b–e, we just show examples of some states along the branches with distinct and significant common features, without implying that all the states along the branches are identical.

**Figure 2.**
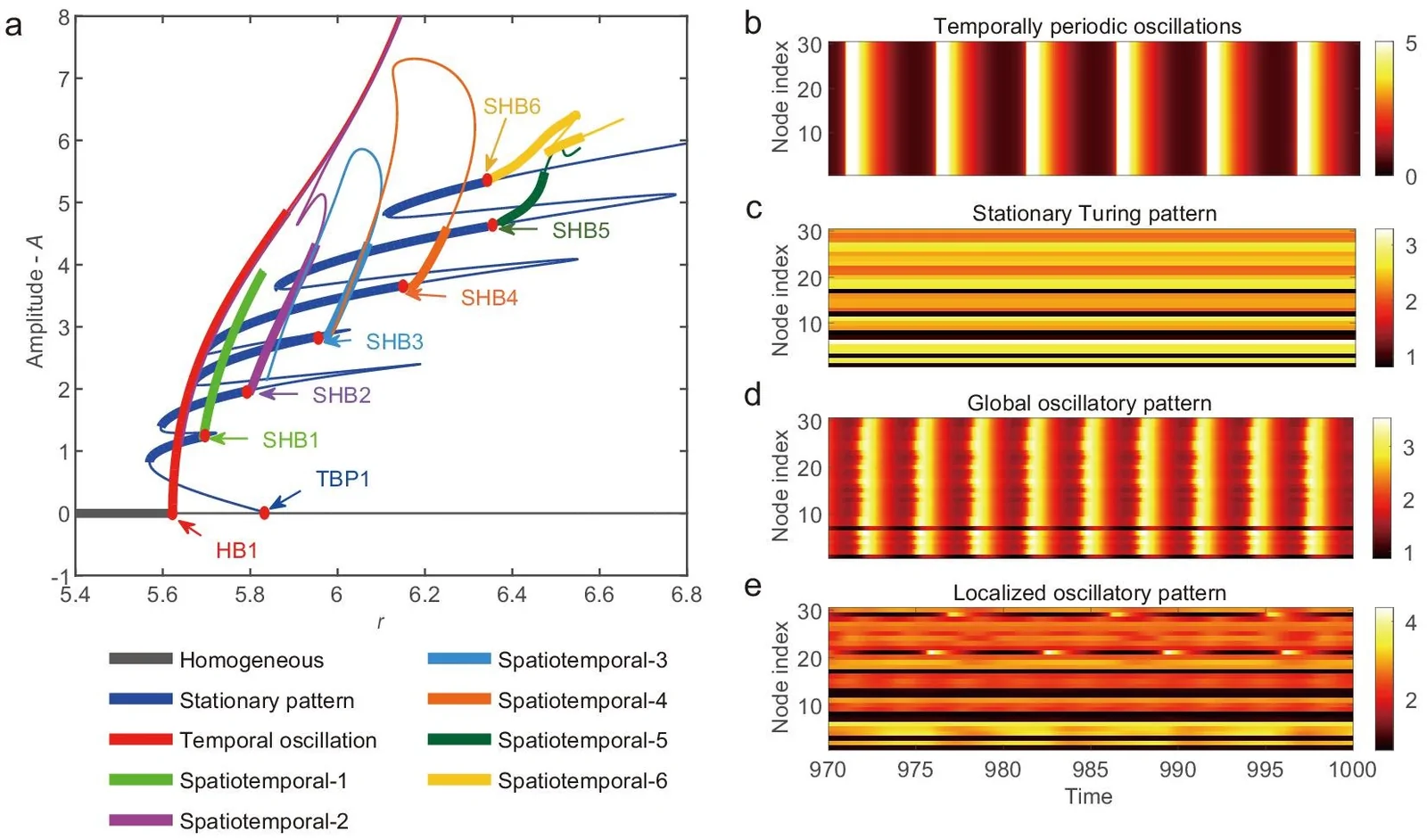
Global branch structure and pattern emergence. (a) The bifurcation diagram of the Brusselator system, showing the amplitude *A*, given by Equation (23), as a function of the control parameter *r*. Thick (thin) lines indicate stable (unstable) solution branches. The bifurcation structure can be interpreted through three successive dynamical regimes. Firstly, the black branch corresponds to the spatially homogeneous steady state, along which the first Hopf bifurcation (HB1) and the first Turing bifurcation point (TBP1) are marked. The red branch denotes spatially homogeneous periodic oscillations emerging from HB1, with a representative state shown in panel (b), characterized by an oscillation index of *R=1* in Equation (26). Secondly, the blue branch represents the subcritical Turing branch (snaking structure), composed of multiple localized steady-state patterns; one representative example is shown in panel (c), corresponding to a non-oscillatory state with *R=0*. Finally, the remaining colored branches correspond to spatiotemporal patterns generated through secondary Hopf bifurcations on the subcritical Turing branch. In particular, branches labeled Spatiotemporal-1 to Spatiotemporal-4 give rise to globally oscillatory patterns, illustrated in panel (d), where *R=1*; whereas Spatiotemporal-5 and Spatiotemporal-6 produce localized oscillatory patterns, illustrated in panel (e), where oscillations are localized on nodes 21 and 28 and *R=2/30≈ 0.06667*. The color bars in panels (b–e) represent the density of the variable $u_i$. In all panels, *ɛ =0.055*, *σ =2.4*, and *p=2.15*.

Further increasing *r* leads to a Turing bifurcation point (TBP1) at $r_T \approx 5.84$, where a subcritical Turing branch composed of numerous stationary patterns appears. The transition from HB1 to TBP1 marks a crucial regime shift where the system progresses from purely temporal oscillations toward space-dependent, diffusion-driven instabilities. One of the corresponding representative states is shown in Fig. 2c with *R=0*. As *r* varies along this branch, it undergoes a sequence of fold points, forming a classical snaking structure and hysteresis loops [56–58]. Note that the existence of this structure requires that the Turing bifurcation is subcritical.

As the parameter *r* now enters the Hopf instability region illustrated in Fig. 1, we detect a series of Hopf bifurcation points (SHB1–SHB6) along the subcritical Turing branch, marking the loss of stability of the stationary Turing patterns. These bifurcations arise from the interaction between the underlying Turing pattern and the Hopf instability, causing the originally stationary spatial patterns to undergo temporal oscillations. Consequently, distinct spatiotemporal states emerge from these bifurcation points. The first four of them are global oscillatory patterns, with one example shown in Fig. 2d with (*R=1*). In these states, all the nodes oscillate with similar periods, while retaining a spatially heterogeneous distribution. In contrast, the last two patterns are localized oscillatory patterns, of which one example is illustrated in Fig. 2e. In these states, only a small subset of nodes, such as nodes 21 and 28, sustain periodic oscillations, while most nodes exhibit only weak fluctuations or remain nearly stationary with (*0< R=0.06667< 1*).

To elucidate the relation between the stationary patterns along the snaking structure and the spatiotemporal patterns on the new branches reported in Fig. 2, first consider the steady-state patterns near the Hopf bifurcation points SHB1–SHB6 (Fig. 3a1–f1). It indicates that, as the number of differentiated nodes increases, the pattern gradually evolves toward a fully developed Turing pattern, similarly to the successive addition of wavelengths in continuous systems, eventually leading to a periodic state [56]. Specifically, as the solution branch traverses successive left folds of the snaking structure, one additional differentiated node is incorporated at each left fold. The parameter interval enclosed by each pair of adjacent fold points defines the existence range of the corresponding localized state. Also, the spatiotemporal patterns inherit the spatial structure of their steady-state counterparts, but acquire oscillatory dynamics due to the Hopf instability. For the first four patterns, all nodes, including the few differentiated ones, exhibit strong, synchronized oscillations. Conversely, as the last two patterns approach a fully developed Turing state, oscillations persist only in a very small subset of nodes, such as node 20 alone, thereby producing localized oscillatory patterns.

**Figure 3.**
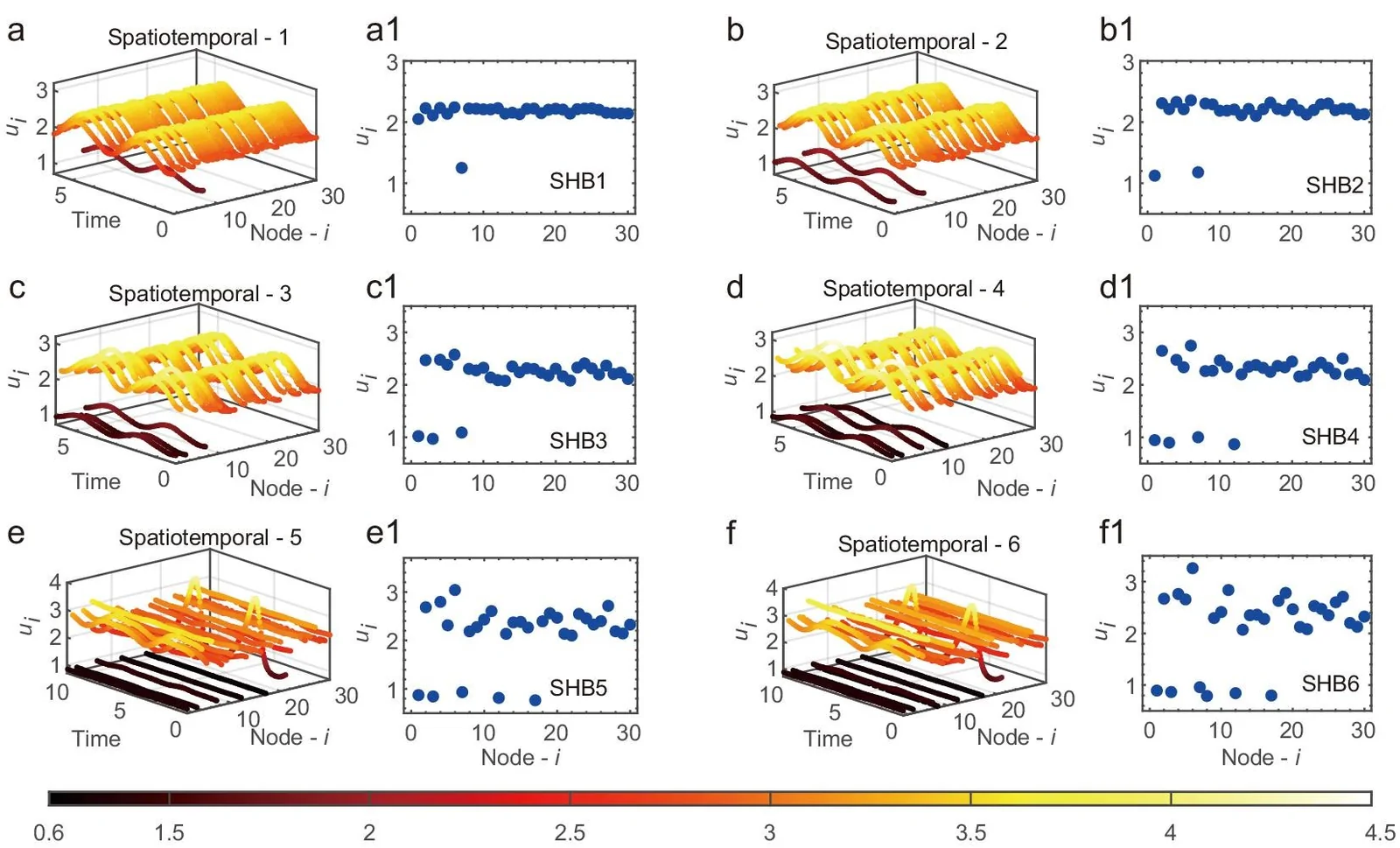
Progressive node differentiation in spatiotemporal patterns. (a–f) Spatiotemporal patterns corresponding to the newly emerged branches. (a1–f1) The corresponding stable steady-state localized patterns at the bifurcation points (SHB1–SHB6). Each time the snaking structure shown in Fig. 2 passes a fold, one more differentiated node is added to the stable steady-state localized pattern. In order, these are nodes 7, 1, 3, 12, 17, and 8. (a–f) Visualizations of the spatiotemporal patterns emerging from the new branches show that the first four patterns are typical globally oscillating patterns, with all nodes oscillating coherently with similar periods, whereas the last two patterns are localized oscillations involving only a subset of nodes (specifically node 20). For each spatiotemporal plot, the color bars are identical to the vertical axes, both of which quantify the density of $u_i$.

### Mechanism validation

To verify that the phenomena described above and observed on BA networks are not specific to this network topology, we study the Brusselator also on Erdős–Rényi random graphs (ER) [61] and Watts–Strogatz small-world networks (WS) [62]. In both cases, we build networks of 30 nodes. For ER graphs, we use connection probability 0.3; for the WS networks, we connect each node to its 8 nearest neighbors, and rewire with probability 0.3. All other parameters are chosen consistently with the results shown above and reported in Fig. 2.

The results, illustrated in Fig. 4, show that varying the network structure induces no significant change in the observed bifurcation behavior, highlighting its robustness across types of network. Specifically, we observe that, as the control parameter *r* is increased, the system undergoes a Hopf bifurcation followed by a Turing one. Along the snaking structure evolving from the Turing bifurcation, additional Hopf bifurcations emerge, giving rise to global oscillatory patterns as well as localized oscillatory patterns. Thus, in both ER graphs (Fig. 4a) and WS networks (Fig. 4b), the branch structures exhibit essentially the same behavior as those obtained on BA networks. The details of the spatiotemporal patterns associated with these branches are also consistent with those shown in Fig. 2, and are therefore not displayed again here.

**Figure 4.**
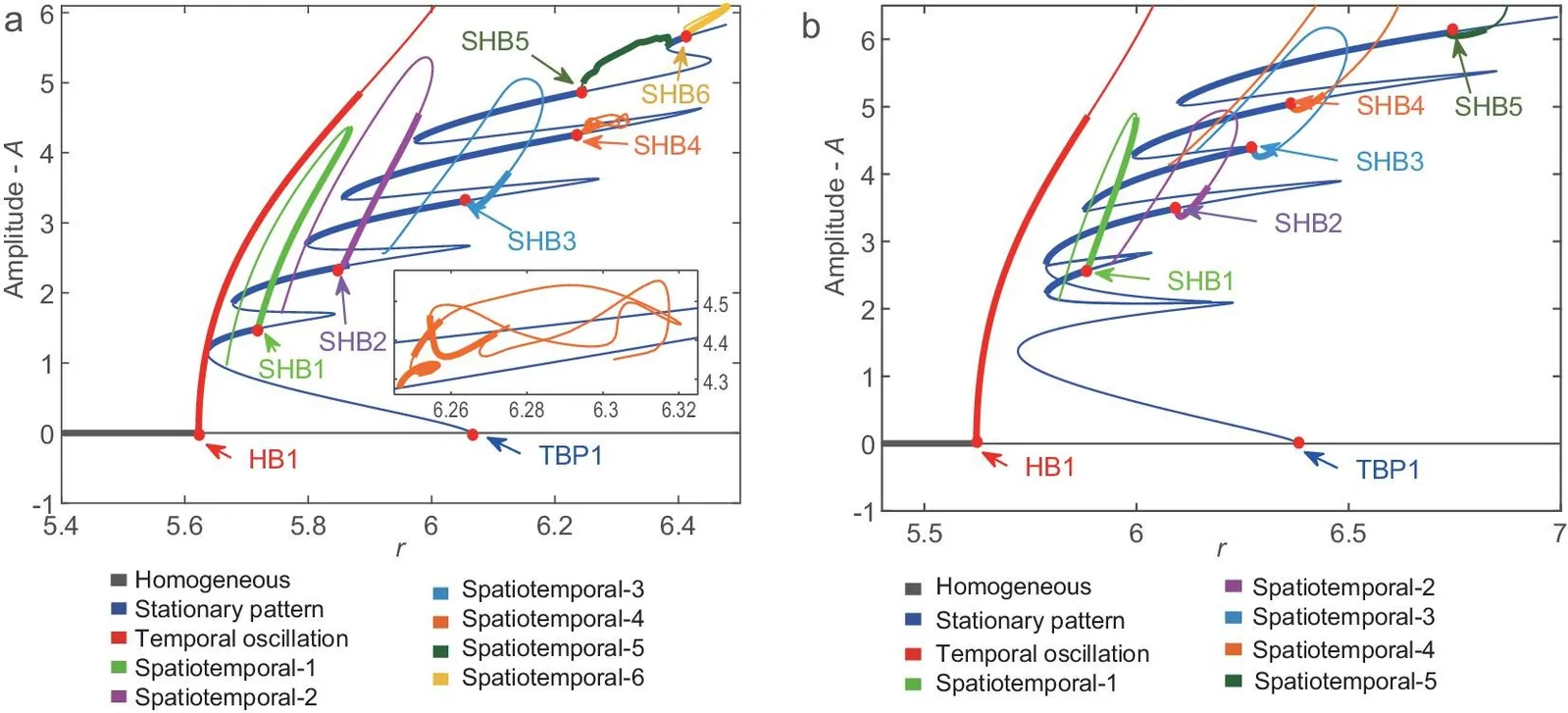
Robustness of pattern emergence with respect to network type. The branch structures on ER random graphs (a) and WS small-world networks (b) are consistent with those obtained on a BA network and shown in Fig. 2. Both networks were generated with 30 nodes. The ER graph has connection probability 0.3; each node in the WS network is connected to 8 nearest neighbors and the rewiring probability is 0.3. The remaining parameters were *ɛ = 0.055*, *σ = 2.4,* and *p = 2.15*.

We further confirm that this phenomenology persists in larger networks, as illustrated in Fig. 5 for BA, ER, and WS networks with *N=1000*. In fact, the emergence of localized oscillatory patterns is most pronounced in the BA network, where oscillatory nodes are fewer but display larger amplitudes due to pronounced degree heterogeneity. In contrast, the ER and WS networks, characterized by relatively uniform degree distributions, exhibit a more homogeneous distribution of oscillatory nodes. One possible explanation for this phenomenon is that high-degree nodes experience stronger diffusive coupling, which stabilizes them near the steady state, whereas weakly connected, low-degree nodes are more susceptible to Hopf oscillations, leading to localized oscillatory patterns. Moreover, Material S3 presents results for BA networks with different sizes (*N=100*, 500, and 5000), all of which exhibit the same qualitative localized oscillatory structures, demonstrating that the underlying mechanism is robust with respect to network size.

**Figure 5.**
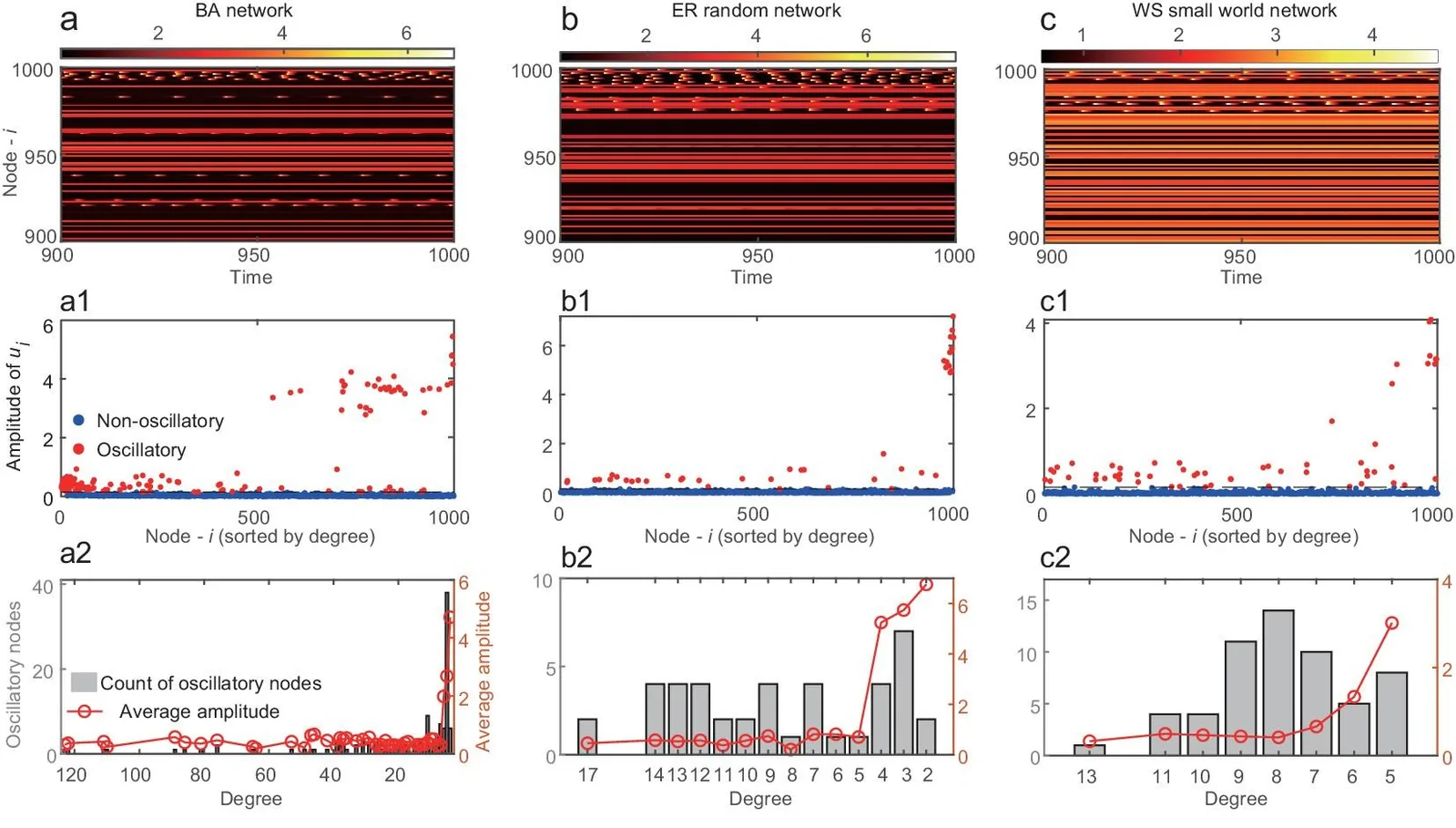
Robustness of pattern emergence with respect to network size. (a–c) Localized oscillatory patterns emerge also in larger networks of 1000 nodes, regardless of the type, as they appear in BA networks (a), ER random graphs (b), and WS small-world networks (c). The patterns are visualized in the top row, where the nodes are sorted in non-increasing order of degree. The color bars represent the density of the state variable $u_i$. The initial clique in the BA network is of size 5, the connection probability in the ER graph is 0.01, and the nodes in the WS network are connected to their 8 nearest neighbours and rewired with probability 0.03. All parameters except *r* are identical for the three cases, and they are *ɛ =0.055*, *σ =2.4,* and *p=2.15*. The values of *r* are 7.8 in panel (a), 7.5 in panel (b) and 6.8 in panel (c). (a1–c1) For all types of networks, nodes with large oscillation amplitude have mostly low degrees, whereas high-degree nodes exhibit only weak or negligible oscillations. (a2–c2) The number of oscillatory nodes (bars, left axis) and the mean amplitude of node oscillations (line, right axis) as a function of degree confirm the results of (a1–c1).

Next, to further validate the proposed mechanism, we examine two additional sets of parameters. First, we put *p = 2.22*, *σ = 2.6*, and *ɛ = 0.055*. In this case, the coefficients in Equation (21), computed following the procedure in Material S1, yield the equation


(27) \begin{eqnarray*}
\frac{\mathrm{d}}{\mathrm{dt}}z &=& 0.69392\mu z + 0.54258z^2 \\
&&+\, 0.011227\mu z^2 + 0.20428z^3,
\end{eqnarray*}

which again indicates a subcritical Turing bifurcation, consistently with the unstable branch observed in AUTO-07P and shown in Fig. 6a. The Hopf instability acting on this subcritical branch at the Hopf bifurcation point HB2 on the snaking structure leads to the emergence of localized oscillatory patterns, illustrated in Fig. 6a1. Conversely, setting *ɛ =0.8* and leaving *p* and *σ* unchanged, we obtain


(28) \begin{eqnarray*}
\frac{\mathrm{d}}{\mathrm{dt}}z &=& 0.71852\mu z - 0.10631 z^2 \\
&&-\, 1.776\mu z^2 - 0.74658 z^3,
\end{eqnarray*}

which indicates that the Turing bifurcation is supercritical, as shown in Fig. 6b. Here, the Hopf instability interacts with the supercritical branch, resulting only in global oscillatory patterns, as seen in Fig. 6b1. This comparison highlights that the subcriticality of the Turing branch is essential for the formation of localized oscillatory patterns.

**Figure 6.**
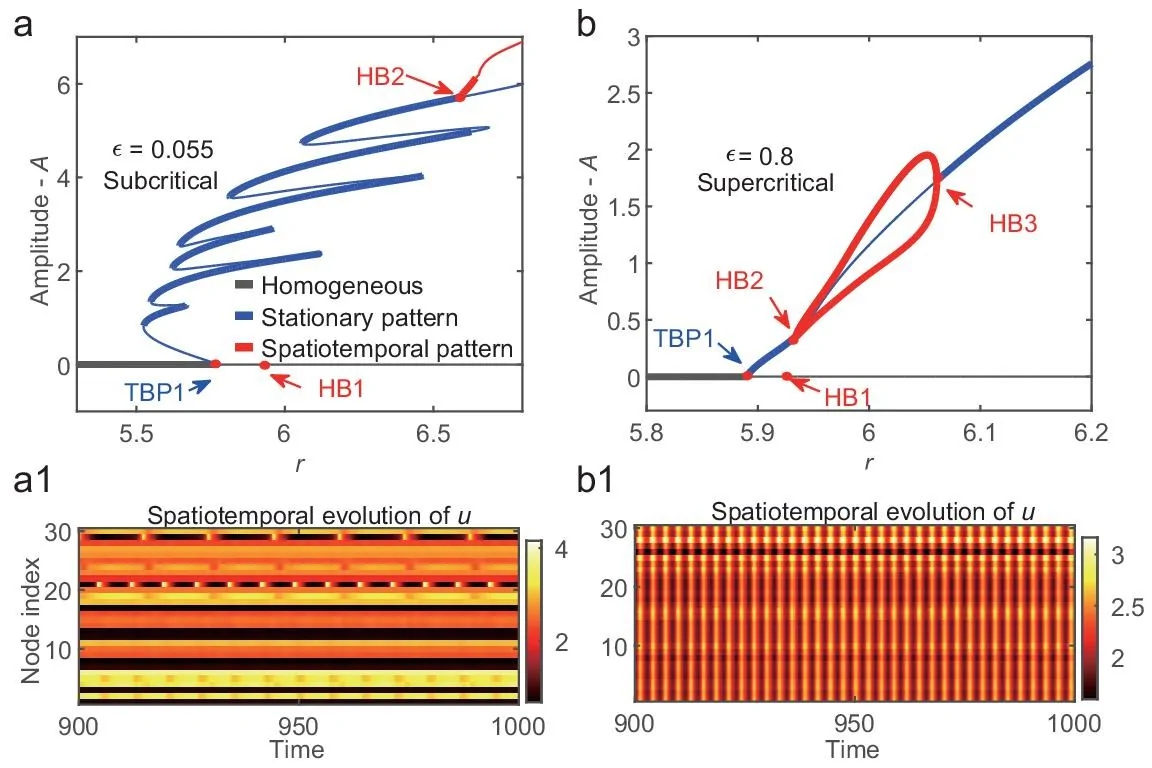
Validation of the mechanism of emergence of local oscillatory patterns. (a) When the parameters of the system are *p=2.22*, *σ =2.6,* and *ɛ =0.055*, a subcritical Turing branch (blue line) appears first, followed by a Hopf bifurcation (HB1). Although this sequence is opposite to that in Fig. 2, the localized oscillatory pattern visualized in (a1) still arises from a second Hopf bifurcation point (HB2) on the subcritical branch, with an IPR value of 0.6906 in Equation (S38), indicating that the associated critical eigenvector is strongly localized. (b) Changing *ɛ* to 0.8, the Turing branch (blue line) becomes supercritical, and the subsequent Hopf bifurcations arising from it (HB2 and HB3) give rise to a global oscillatory pattern, shown in (b1), associated with a much smaller IPR value (0.133), reflecting a delocalized state.

This transition is also accompanied by a marked change in the localization of the critical Turing eigenmode, quantified by the inverse participation ratio (IPR; Equation (S38)), with larger IPR values indicating stronger spatial localization. For the subcritical case, the IPR reaches 0.6909, reflecting a highly localized critical eigenmode that is consistent with the emergence of localized patterns. In contrast, the supercritical case yields a much smaller IPR of 0.1333, indicating a delocalized eigenmode consistent with the extended spatial pattern.

These two cases further suggest that localized oscillatory patterns disappear as the Turing bifurcation changes from subcritical to supercritical with increasing *ɛ*. This naturally raises the question of the parameter range over which localized oscillatory patterns can exist. To address this issue, Material S4 systematically determines the critical interval of *ɛ* supporting localized oscillatory states. Together, these results demonstrate that localized oscillatory patterns specifically originate from the Hopf instability acting on a subcritical Turing branch.

Finally, to show that the mechanism is robust also with respect to changes in the specific system, we analyzed one more reaction–diffusion model, namely the FHN model [63]. Originally introduced to study neuronal excitability and action potential propagation, the FHN model is now widely employed to investigate pattern formation in spatially extended systems [64]. In this model, the local reaction kinetics is given by


(29) \begin{eqnarray*}
\mathbf {F}(\mathbf {U}_i) =
\left(\begin{array}{c}
{u_i - u_i^3 - v_i}\\
{\varepsilon \left(u_i-av_i-b\right)}\end{array}\right),
\end{eqnarray*}

where *a, b*, and *ɛ* are positive parameters and *i=1,… ,N*. The elements of the diffusion matrix are $d_u$ and $d_v$, allowing the diffusion rates of the two components to be varied independently. For convenience, we define the amplitude in the bifurcation diagrams as


(30) \begin{eqnarray*}
A = \sqrt{\sum _{i=1}^N \left(u_i(\mathrm{T})^2 + v_i(\mathrm{T})^2 \right)},
\end{eqnarray*}

since obtaining a general analytical expression for the positive equilibrium is quite difficult.

In Fig. 7, we report the branch structures and localized oscillatory patterns of the FHN model on BA networks, ER graphs and WS small-world networks, generated using the same procedures as for the Brusselator. We observe that the subcritical branch gives rise to new spatiotemporal patterns through Hopf instabilities. The observation of the same bifurcation structure and localized oscillatory states in a network FHN system demonstrates that the proposed mechanism is not tied to the specific nonlinear structure of the Brusselator model. Rather, it reflects a generic mechanism associated with instability-driven pattern formation in network reaction–diffusion systems.

**Figure 7.**
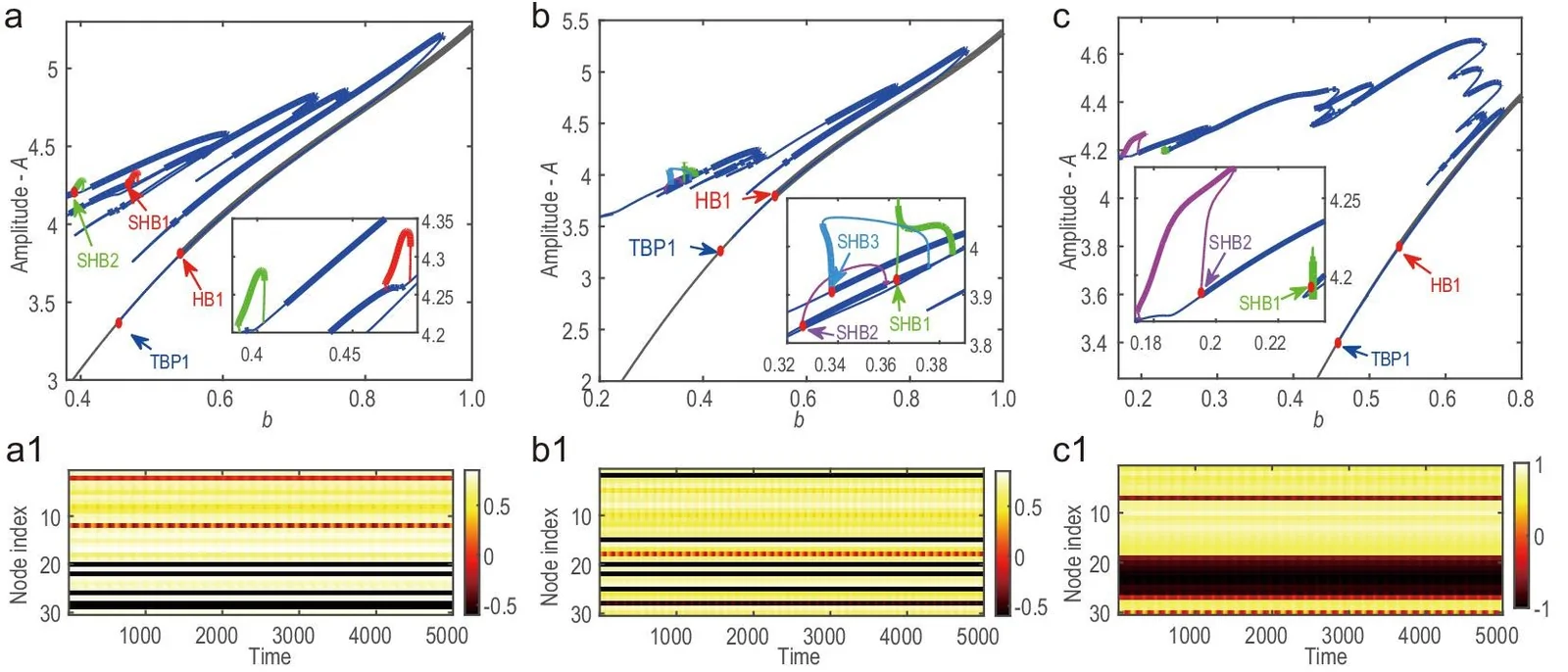
Robustness of pattern emergence with respect to system. (a–c) The branch structures of the FitzHugh–Nagumo (FHN) model on BA networks (a), ER random graphs (b), and WS small-world networks (c). Across the three different network topologies, qualitatively similar bifurcation processes are observed: the subcritical Turing branches (blue lines) further undergo secondary Hopf bifurcations (SHB1–SHB3), giving rise to spatiotemporal branches (colored curves) corresponding to localized oscillatory patterns. This result demonstrates that the proposed bifurcation mechanism is not restricted to the Brusselator system but can be successfully reproduced in the FHN model with distinct local dynamics. The insets provide magnified views of the parameter regions where these localized oscillatory states emerge to highlight the detailed structures. (a1–c1) Spatiotemporal evolution diagrams of representative localized oscillatory states on the branches corresponding to SHB1–SHB3. It is evident that the oscillatory activity is primarily confined to a small subset of nodes, while the vast majority of nodes remain nearly stationary. In all panels, *b* is used as the continuation parameter, and the other parameter values are fixed at *a=0.1*, *ɛ =0.01*, $d_u=0.1$, and $d_v=1.0$.

## CONCLUSIONS

In this work, we propose an alternative mechanism for the emergence of localized oscillatory patterns in networked two-dimensional reaction–diffusion systems, namely Hopf-type instabilities acting on a subcritical Turing branch. This mechanism overcomes the restriction of the classical wave bifurcation (oscillatory Turing instability), which requires at least three interacting components. Combining normal-form analysis with numerical continuation, we uncover the underlying branch structure and reveal the transition from global oscillations to localized patterns as system parameters vary. Moreover, we demonstrate that the emergence of such patterns is robust across different network sizes and types, as well as different reaction–diffusion systems.

Notably, the normal form coefficients we generate are more than just determinants of the bifurcation type. Rather, they are directly related to the topological heterogeneity of the network via the spectral decomposition inherent in our analysis. The critical cubic coefficient $C_{30}$, which dictates subcriticality ($C_{30}> 0$), explicitly incorporates the fourth spectral moment $S_4^{(T)}$ via the term $a_{300} = \mathbf {v}_T^{*} \mathbf {Q}_{300} S_4^{(T)}$ in Equation (S38). On a 1000-node regular lattice (with 8 nearest neighbors), the delocalized eigenvectors ($\phi _i \sim O(N^{-1/2})$) yield $S_3^{(T)} = 0$ and a vanishingly small $S_4^{(T)} = 0.0015 \approx 1/N$. Conversely, topological heterogeneity induces severe eigenvector localization, concentrating the critical mode amplitude ($\phi _i \sim O(1/\sqrt{K})$) onto a small active subset of *K* nodes, while leaving the remaining nodes near zero. Under this localized state, the fourth moment scales as $S_4^{(T)} \sim O(1/K)$, which remains non-vanishingly large in the thermodynamic limit. For the three *N=1000* network topologies shown in Fig. 5, our calculations yield $S_4^{(T)} = 0.6638$ (BA), 0.7482 (ER), and 0.5650 (WS), while $S_3^{(T)}$ takes the values *-0.6116*, 0.7939, and *-0.6244*, respectively. This quantitative jump, which amplifies $S_4^{(T)}$ by more than 300 times compared with the regular lattice, drastically enhances the direct cubic projection term $a_{300}$. Such a substantial order-of-magnitude discrepancy in the spectral moments is sufficient to reverse the sign of $C_{30}$. The emergence of localized oscillatory patterns is therefore not an intrinsic property of the model itself, but rather a direct consequence of the interplay between nonlinear dynamics and the spectral signature of topological heterogeneity.

Also, the emergence of localized oscillatory patterns marks a transition from global synchrony [65,66] to asynchronous dynamics amongst network nodes, indicating that a single system can intrinsically support a diversity of spatiotemporal states, including complete synchrony, strong disorder and partial synchrony. From an applied perspective, this intrinsic multistability may offer a theoretical basis for future strategies to sustain healthy synchrony or suppress pathological synchronization, such as cognitive performance [67] and abnormal neural synchrony in Parkinson’s disease and epilepsy [68]. Moreover, our findings may provide new perspectives for understanding localized spreading phenomena in heterogeneous networked systems. For example, in epidemiological networks, the combined effects of topological heterogeneity, eigenvector localization, and uneven intervention intensity may naturally induce localized outbreak dynamics confined to specific regions or subnetworks [69]. Therefore, clarifying the formation mechanisms and bifurcation structures of localized steady and oscillatory patterns may help reveal the dynamical origin of recurrent regional outbreaks, and further provide theoretical guidance for the development of targeted intervention and control strategies in realistic spreading systems.

## Supplementary Material

nwag523_Supplemental_File
