# Supplementary material for: An alternate mechanism for the emergence of localized oscillatory patterns in networked reaction–diffusion systems
Source: Natl Sci Rev. 2026 Sep 2;13(19):nwag523. doi: 10.1093/nsr/nwag523 (PMC13637610; doi:10.1093/nsr/nwag523)
Supplement: Supplementary file 1 [file nwag523_supplemental_file.pdf]

Supplementary Material for

An alternate mechanism for the emergence of localized oscillatory patterns in networked reaction–diffusion systems

Zhi-Chao Xue[1,†], Xiaofeng Luo[2,3,†], Li Li[4,†], Yongli Song[5], Stefano Boccaletti[3,6,7], Charo I. del Genio[6,8,*], Yi-Cheng Zhang[3,9,*], Gui-Quan Sun[2,3,1,*]

[1]Complex Systems Research Center, Shanxi University, Taiyuan 030006, China
[2]School of Mathematics, North University of China, Taiyuan 030051, China
[3]Sino-Europe Complex Science Center, North University of China, Taiyuan 030051, China
[4]School of Computer and Information Technology, Shanxi University, Taiyuan 030006, China
[5]School of Mathematics, Hangzhou Normal University, Hangzhou 311121, China
[6]Institute of Interdisciplinary Intelligent Science, Ningbo University of Technology, Ningbo, China
[7]CNR-Institute of Complex Systems, Via Madonna del Piano 10, I-50019 Sesto Fiorentino, Italy
[8]Bulgarian Academy of Sciences, Institute of Biophysics and Biomedical Engineering, ul. Acad. Georgi Bonchev 21, Sofia 1113, Bulgaria
[9]Physics Department, University of Fribourg Chemin du Musée 3, 1700 Fribourg, Switzerland

[†]These authors contributed equally to this work
[*]Corresponding authors. Email: charo.delgenio@trakia-uni.bg (C.I. del Genio), zinppy@gmail.com (Y.-C. Zhang), gquansun@126.com (G.-Q. Sun)

This Supplementary Information provides additional theoretical derivations, numerical methodological details, and robustness analyses supporting the main text.

S1 Derivation of the Reduced Dynamics on the Center Manifold

Here, we derive the reduced dynamics of the Brusselator on the center manifold, consisting of trajectories near the homogeneous steady state that exhibit neither exponential growth nor decay. To this end, we follow the procedure of Ref. [1]. Under the simple critical Laplacian eigenvalue assumption stated in the main text, the center manifold is one-dimensional, and the local dynamics near the bifurcation point can be described by a single scalar amplitude equation. This contrasts with the case of a repeated critical eigenvalue, for which the center manifold becomes higher-dimensional and the dynamics must be described by a coupled system of amplitude equations.

S1.1 State space decomposition

We start by introducing shifted variables $\tilde{u}_i = u_i - u^*$, $\tilde{v}_i = v_i - v^*$ and $\mu = r - r_T$, so that the bifurcation occurs at the origin of the (u, v) phase diagram with $\mu = 0$.

Next, we express the linearized version of the system in Eq. (1) in main text for the entire network as

$$\frac{\partial}{\partial \mathrm{t}}\mathbf{U} = \left(\mathbf{I}_N \otimes J\mathbf{F}|_{\mathbf{0}}\right)\mathbf{U} + \left(\mathbf{L} \otimes \mathbf{D}\right)\mathbf{U}\,, \tag{S1}$$

where $\mathbf{I}_N$ is the N-dimensional identity matrix, $\otimes$ denotes the Kronecker product and we have introduced the $2N$-dimensional vector

$$\mathbf{U} = \left(\mathbf{U}_1^{\mathrm{T}}, \mathbf{U}_2^{\mathrm{T}}, \ldots, \mathbf{U}_N^{\mathrm{T}}\right)^{\mathrm{T}} = \left(\tilde{u}_1, \tilde{v}_1, \ldots, \tilde{u}_N, \tilde{v}_N\right)^{\mathrm{T}}\,. \tag{S2}$$

Calling Λ_T the Laplacian eigenvalue in correspondence of which the system undergoes a bifurcation, and using Eq. (13) and Eq. (15) from the main text into Eq. (S1), we find the following eigenvector equation for the critical mode:

$$\begin{pmatrix} r_T - 1 + \varepsilon\Lambda_T & p^2 \\ -r_T & -p^2 + \varepsilon\sigma\Lambda_T \end{pmatrix} \mathbf{v}_T = \mathbf{0}\,. \tag{S3}$$

Putting $\mathbf{v}_T = (a_T, b_T)^{\mathrm{T}}$, and choosing the normalization $b_T = 1$, the solution to the equation above is

$$a_T = \frac{-p^2}{r_T - 1 + \varepsilon\Lambda_T}\,. \tag{S4}$$

Similarly, we can find the left eigenvector $\mathbf{v}_T^* = (a_T^*, b_T^*)^{\mathrm{T}}$ of the matrix in Eq. (S3), which, upon imposing the normalization $\mathbf{v}_T^* \cdot \mathbf{v}_T = 1$, is given by

$$a_T^* = \frac{r_T\,(r_T - 1 + \varepsilon\Lambda_T)^2}{(r_T - 1 + \varepsilon\Lambda_T)\left[(r_T - 1 + \varepsilon\Lambda_T)^2 - p^2 r_T\right]} \tag{S5}$$

$$b_T^* = \frac{(r_T - 1 + \varepsilon\Lambda_T)^2}{(r_T - 1 + \varepsilon\Lambda_T)^2 - p^2 r_T}. \tag{S6}$$

Finally, we define the vectors $\mathbf{q}$ and $\mathbf{q}^*$ as

$$\mathbf{q} = \boldsymbol{\phi}^{(T)} \otimes \mathbf{v}_T \tag{S7}$$

$$\mathbf{q}^* = \boldsymbol{\phi}^{(T)} \otimes \mathbf{v}_T^*\,, \tag{S8}$$

where $\boldsymbol{\phi}^{(T)}$ is the Laplacian eigenvector corresponding to the eigenvalue Λ_T. These vectors allow us to describe the center subspace T_c as the subspace spanned by the vector $\mathbf{q}$, and the subspace T_{su} as its complement. In formulae,

$$T_c = \{z\mathbf{q}\} \quad \forall z \in \mathbb{R} \tag{S9}$$

$$T_{su} = \{\mathbf{U} \mid (\mathbf{q}^* \cdot \mathbf{U}) = 0\}\,. \tag{S10}$$

S1.2 Center manifold construction

To construct the center manifold we first separate the dynamics between the center subspace and the complementary one, by decomposing any state $\mathbf{U}$ as $\mathbf{U} = z\mathbf{q} + \mathbf{w}$. From the treatment in the previous subsection, we get

$$z = \mathbf{q}^* \cdot \mathbf{U} \tag{S11}$$

$$\mathbf{w} = \mathbf{U} - (\mathbf{q}^* \cdot \mathbf{U})\,\mathbf{q}\,, \tag{S12}$$

where $\mathbf{q}$ and $\mathbf{q}^*$ are given by Eqs. (S7) and (S8), respectively.

Now, consider the differential operator in the right-hand side of Eq. (S1) and note that it implicitly depends on the value of μ. We can make this dependence explicit by defining

$$\mathcal{E}_\mu = \mathbf{I}_N \otimes J\mathbf{F}|_{\mu,\mathbf{0}} + \mathbf{L} \otimes \mathbf{D}\,. \tag{S13}$$

In particular, $\mathcal{E}_0 = \mathbf{I}_N \otimes J\mathbf{F}|_{0,\mathbf{0}} + \mathbf{L} \otimes \mathbf{D}$ denotes the operator at the bifurcation point $\mu = 0$. Using $\mathcal{E}_0$ as the reference operator, the system in Eq. (1) in main text can be rewritten as

$$\frac{\partial}{\partial \mathrm{t}}\mathbf{U} = \mathcal{E}_0\mathbf{U} + \mathcal{N}_\mu(\mathbf{U}). \tag{S14}$$

where

$$\mathcal{N}_\mu = \mathbf{F}(\mathbf{U}, \mu) - (\mathbf{I}_N \otimes J\mathbf{F}|_{0,\mathbf{0}})\mathbf{U}. \tag{S15}$$

This form is particularly useful because $\mathcal{E}_0\mathbf{q} = \mathcal{E}_0^{\mathrm{T}}\mathbf{q}^* = \mathbf{0}$. Then, we can rewrite the evolution of the system in terms of those of z and $\mathbf{w}$ as

$$\frac{\mathrm{d}}{\mathrm{dt}}z = \mathbf{q}^* \cdot \mathcal{F}_\mu(z\mathbf{q} + \mathbf{w}) \tag{S16}$$

$$\frac{\mathrm{d}}{\mathrm{dt}}\mathbf{w} = \mathcal{E}_0\mathbf{w} + \mathcal{F}_\mu(z\mathbf{q} + \mathbf{w}) - (\mathbf{q}^* \cdot \mathcal{F}_\mu(z\mathbf{q} + \mathbf{w}))\,\mathbf{q}\,. \tag{S17}$$

Next, we expand $\mathcal{F}_\mu$ up to third order around the origin. To simplify the treatement, we proceed systematically, order by order, and we write the two components of **F** as

$$f(\tilde{u}_i, \tilde{v}_i, \mu) = p - (\mu + r_T + 1)(\tilde{u}_i + u^*) + (\tilde{u}_i + u^*)^2 (\tilde{v}_i + v^*) \tag{S18}$$

$$g(\tilde{u}_i, \tilde{v}_i, \mu) = (\mu + r_T) - (\tilde{u}_i + u^*)^2 (\tilde{v}_i + v^*) \ , \tag{S19}$$

where $i = 1, \ldots, N$. Then, the second-order terms in the expansion of f are

$$f^{(2)}(\tilde{u}_i, \tilde{v}_i, \mu) = \frac{1}{2}\left(f_{200}\tilde{u}_i^2 + 2f_{110}\tilde{u}_i\tilde{v}_i + f_{020}\tilde{v}_i^2\right) + f_{101}\mu\tilde{u}_i + f_{011}\mu\tilde{v}_i \tag{S20}$$

and the third-order ones are

$$f^{(3)}(\tilde{u}_i, \tilde{v}_i, \mu) = \frac{1}{6}f_{300}\tilde{u}_i^3 + \frac{1}{2}f_{210}\tilde{u}_i^2\tilde{v}_i + \frac{1}{2}f_{120}\tilde{u}_i\tilde{v}_i^2 + \frac{1}{6}f_{030}\tilde{v}_i^3 + \frac{1}{2}f_{201}\mu\tilde{u}_i^2 + f_{111}\mu\tilde{u}_i\tilde{v}_i + \frac{1}{2}f_{021}\mu\tilde{v}_i^2 \ , \tag{S21}$$

where we used the shorthand

$$f_{ijk} = \left.\frac{\partial^{i+j+k}}{\partial\tilde{\mathrm{u}}^i\partial\tilde{\mathrm{v}}^j\partial\mu^k} f(\tilde{u}, \tilde{v}, \mu)\right|_{(0,0,0)} \ . \tag{S22}$$

Similarly, we obtain

$$g^{(2)}(\tilde{u}_i, \tilde{v}_i, \mu) = \frac{1}{2}\left(g_{200}\tilde{u}_i^2 + 2g_{110}\tilde{u}_i\tilde{v}_i + g_{020}\tilde{v}_i^2\right) + g_{101}\mu\tilde{u}_i + g_{011}\mu\tilde{v}_i \tag{S23}$$

and

$$g^{(3)}(\tilde{u}_i, \tilde{v}_i, \mu) = \frac{1}{6}g_{300}\tilde{u}_i^3 + \frac{1}{2}g_{210}\tilde{u}_i^2\tilde{v}_i + \frac{1}{2}g_{120}\tilde{u}_i\tilde{v}_i^2 + \frac{1}{6}g_{030}\tilde{v}_i^3 + \frac{1}{2}g_{201}\mu\tilde{u}_i^2 + g_{111}\mu\tilde{u}_i\tilde{v}_i + \frac{1}{2}g_{021}\mu\tilde{v}_i^2 \ , \tag{S24}$$

where, in analogy to f_{ijk}, g_{ijk} denotes the $(i+j+k)$-th mixed partial derivative of g with respect to the i-th power of $\tilde{u}_i$, the j-th power of $\tilde{v}_i$ and the k-th power of μ, evaluated at the origin.

Now note that, from the decomposition of **U**, we get that, for each node i,

$$\tilde{u}_i = z a_T \phi_i^{(T)} + w_{2i-1} \tag{S25}$$

$$\tilde{v}_i = z b_T \phi_i^{(T)} + w_{2i} \ . \tag{S26}$$

We can then use these equations to compute expressions for the second-order functions of $\tilde{u}_i$, $\tilde{v}_i$ and μ in Eq. (S20), obtaining:

$$\tilde{u}_i^2 \approx z^2 a_T^2 \left(\phi_i^{(T)}\right)^2 + 2z a_T \phi_i^{(T)} w_{2i-1} \tag{S27}$$

$$\tilde{v}_i^2 \approx z^2 b_T^2 \left(\phi_i^{(T)}\right)^2 + 2z b_T \phi_i^{(T)} w_{2i} \tag{S28}$$

$$\tilde{u}_i\tilde{v}_i \approx z^2 a_T b_T \left(\phi_i^{(T)}\right)^2 + z\phi_i^{(T)}\left(a_T w_{2i} + b_T w_{2i-1}\right) \tag{S29}$$

$$\mu\tilde{u}_i \approx \mu z a_T \phi_i^{(T)} + \mu w_{2i-1} \tag{S30}$$

$$\mu\tilde{v}_i \approx \mu z b_T \phi_i^{(T)} + \mu w_{2i} \ . \tag{S31}$$

Note that the variables **w** represent higher-order corrections associated with the stable modes on the center manifold. In particular, they are slaved to the critical mode amplitude z and satisfy $\mathbf{w} = \mathcal{O}(z^2, \mu z)$. Therefore, when collecting second-order contributions in the reduced expansion, we first retain only the terms purely quadratic in z and the mixed terms of order μz. The contributions involving w are treated separately as higher-order terms and will be handled subsequently. Consider now only the terms in z^2 and μz in the expressions above. Their contribution, when substituting them into Eq. (S20) and Eq. (S23), is

$$\frac{z^2}{2}\left(\phi_i^{(T)}\right)^2\left(a_T^2\begin{pmatrix}f_{200}\\g_{200}\end{pmatrix} + 2a_T b_T\begin{pmatrix}f_{110}\\g_{110}\end{pmatrix} + b_T^2\begin{pmatrix}f_{020}\\g_{020}\end{pmatrix}\right) + \mu z\phi_i^{(T)}\left(a_T\begin{pmatrix}f_{101}\\g_{101}\end{pmatrix} + b_T\begin{pmatrix}f_{011}\\g_{011}\end{pmatrix}\right) \ . \tag{S32}$$

Thus, globally, the contribution of these terms within $\mathcal{F}_\mu\mathbf{U}$ can be written as a vector consisting of N two-dimensional vectors such that the i-th such vector is given by Eq. (S32).

Also, using Eq. (S8), **the contribution of these terms to the scalar product between $\mathbf{q}^*$ and $\mathbf{F}_\mu \mathbf{U}$ is**

$$\frac{z^2}{2}\mathbf{v}_T^{*\,\mathrm{T}}\left(a_T^2\begin{pmatrix}f_{200}\\g_{200}\end{pmatrix}+2a_Tb_T\begin{pmatrix}f_{110}\\g_{110}\end{pmatrix}+b_T^2\begin{pmatrix}f_{020}\\g_{020}\end{pmatrix}\right)\sum_{i=1}^{N}\left(\phi_i^{(T)}\right)^3 + \mu z\mathbf{v}_T^{*\,\mathrm{T}}\left(a_T\begin{pmatrix}f_{101}\\g_{101}\end{pmatrix}+b_T\begin{pmatrix}f_{011}\\g_{011}\end{pmatrix}\right)\sum_{i=1}^{N}\left(\phi_i^{(T)}\right)^2 , \quad \text{(S33)}$$

which we can write more succinctly as

$$\frac{z^2}{2}\mathbf{v}_T^{*\,\mathrm{T}}\mathbf{Q}_{200}S_3^{(T)} + \mu z\mathbf{v}_T^{*\,\mathrm{T}}\mathbf{Q}_{101}S_2^{(T)} \quad \text{(S34)}$$

by putting

$$\mathbf{Q}_{200} = \begin{pmatrix}a_T^2 f_{200} + 2a_Tb_Tf_{110} + b_T^2 f_{020}\\ a_T^2 g_{200} + 2a_Tb_Tg_{110} + b_T^2 g_{020}\end{pmatrix}, \quad \text{(S35)}$$

$$\mathbf{Q}_{101} = \begin{pmatrix}a_T f_{101} + b_T f_{011}\\ a_T g_{101} + b_T g_{011}\end{pmatrix} \quad \text{(S36)}$$

and

$$S_m^{(T)} = \sum_{i=1}^{N}\left(\phi_i^{(T)}\right)^m . \quad \text{(S37)}$$

Here, $S_m^{(T)}$ represents the higher-order moments of the Laplacian eigenvectors. Note that these spectral moments act as fundamental order parameters for our network system. The first-order moment $S_1^{(T)}$ vanishes due to the standard orthogonality condition of the Laplacian eigenvectors, while the second-order moment $S_2^{(T)}$ is normalized to unity ($S_2^{(T)} = 1$). It is the higher-order moments ($m \geq 3$) that capture the essential, non-Gaussian structure of the eigenvector distribution, which governs the leading-order nonlinear interactions. Thus, higher-order moments control the nonlinear coupling between different Laplacian modes.

In particular, the third and fourth-order moments, $S_3^{(T)}$ and $S_4^{(T)}$, are particularly critical, as they quantify the breaking of topological symmetry and the degree of eigenvector localization inherent to disordered networks. Specifically, the localization of the critical Laplacian mode can be quantified by the inverse participation ratio (IPR) [6],

$$\mathrm{IPR} = \frac{\sum_{i=1}^{N}\left(\phi_i^{(T)}\right)^4}{\left(\sum_{i=1}^{N}\left(\phi_i^{(T)}\right)^2\right)^2}. \quad \text{(S38)}$$

Under the standard eigenvector normalization $S_2^{(T)} = 1$, this reduces to

$$\mathrm{IPR} = S_4^{(T)}. \quad \text{(S39)}$$

For a fully delocalized mode distributed approximately uniformly over all nodes, $\mathrm{IPR} \sim 1/N$, whereas increasingly localized modes yield larger IPR values, approaching 1 in the maximally localized limit. Their non-vanishing values directly incorporate the structural heterogeneity of the network into the normal form coefficients, which govern the nonlinear saturation and ultimately select a subcritical bifurcation pathway conducive to the emergence of localized patterns.

Finally, we can identify the scalar coefficients

$$a_{200} = \mathbf{v}_T^{*\,\mathrm{T}}\mathbf{Q}_{200}S_3^{(T)} \quad \text{(S40)}$$

and

$$a_{101} = \mathbf{v}_T^{*\,\mathrm{T}}\mathbf{Q}_{101}S_2^{(T)} , \quad \text{(S41)}$$

and rewrite the contribution as

$$\frac{z^2}{2}a_{200} + a_{101}\mu z\,. \tag{S42}$$

Consider now the terms in zw and μw in Eqs. (S27)–(S31). Repeating the calculation, one finds that the contribution these terms give to $\mathcal{F}_\mu \mathbf{U}$ can be expressed as

$$\mu\left(\mathbf{a_{011}}\cdot\mathbf{w}\right) + z\left(\mathbf{a_{110}}\cdot\mathbf{w}\right)\,, \tag{S43}$$

where $\mathbf{a_{011}}$ and $\mathbf{a_{110}}$ are $2N$-dimensional vectors whose elements are given, for every node i, by

$$(\mathbf{a_{011}})_{2i-1} = \mathbf{v}_T^*\cdot\begin{pmatrix}f_{101}\\ g_{101}\end{pmatrix}\phi_i^{(T)} \tag{S44}$$

$$(\mathbf{a_{011}})_{2i} = \mathbf{v}_T^*\cdot\begin{pmatrix}f_{011}\\ g_{011}\end{pmatrix}\phi_i^{(T)} \tag{S45}$$

and

$$(\mathbf{a_{110}})_{2i-1} = \mathbf{v}_T^*\cdot\left(\begin{pmatrix}f_{200}\\ g_{200}\end{pmatrix}a_T + \begin{pmatrix}f_{110}\\ g_{110}\end{pmatrix}b_T\right)\left(\phi_i^{(T)}\right)^2 \tag{S46}$$

$$(\mathbf{a_{110}})_{2i} = \mathbf{v}_T^*\cdot\left(\begin{pmatrix}f_{110}\\ g_{110}\end{pmatrix}a_T + \begin{pmatrix}f_{020}\\ g_{020}\end{pmatrix}b_T\right)\left(\phi_i^{(T)}\right)^2\,, \tag{S47}$$

respectively.

Finally, the whole procedure can be carried out on the third-order terms in Eq. (S21) and Eq. (S24). Ultimately, this yields that their contributions to $\mathcal{F}_\mu \mathbf{U}$ are

$$\frac{1}{6}a_{300}z^3 + \frac{1}{2}a_{201}\mu z^2\,. \tag{S48}$$

In this equation, the scalar coefficients are

$$a_{300} = \mathbf{v}_T^*\mathbf{Q}_{300}S_4^{(T)} \tag{S49}$$

and

$$a_{201} = \mathbf{v}_T^*\mathbf{Q}_{201}S_3^{(T)}\,, \tag{S50}$$

where we put

$$\mathbf{Q}_{300} = a_T^3\begin{pmatrix}f_{300}\\ g_{300}\end{pmatrix} + 3a_T^2 b_T\begin{pmatrix}f_{210}\\ g_{210}\end{pmatrix} + 3a_T b_T^2\begin{pmatrix}f_{120}\\ g_{120}\end{pmatrix} + b_T^3\begin{pmatrix}f_{030}\\ g_{030}\end{pmatrix} \tag{S51}$$

and

$$\mathbf{Q}_{201} = a_T^2\begin{pmatrix}f_{201}\\ g_{201}\end{pmatrix} + 2a_T b_T\begin{pmatrix}f_{111}\\ g_{111}\end{pmatrix} + b_T^2\begin{pmatrix}f_{021}\\ g_{021}\end{pmatrix}\,. \tag{S52}$$

These expressions allow one to approximate the system in Eqs. (S16) and (S17) as

$$\frac{\mathrm{d}}{\mathrm{dt}}z \approx \frac{1}{2}a_{200}z^2 + a_{101}\mu z + \mu\left(\mathbf{a_{011}}\cdot\mathbf{w}\right) + z\left(\mathbf{a_{110}}\cdot\mathbf{w}\right) + \frac{1}{6}a_{300}z^3 + \frac{1}{2}a_{201}\mu z^2 \tag{S53}$$

$$\frac{\mathrm{d}}{\mathrm{dt}}\mathbf{w} \approx \mathcal{E}_0\mathbf{w} + \frac{1}{2}\mathbf{b_{200}}z^2 + \mathbf{b_{101}}\mu z\,, \tag{S54}$$

where $\mathbf{b_{200}}$ and $\mathbf{b_{101}}$ are vectors containing N blocks of 2 elements, so that, for each node i, the corresponding blocks are given by

$$(\mathbf{b_{200}})_i = \mathbf{Q}_{200}\left(\phi_i^{(T)}\right)^2 - a_{200}\mathbf{v}_T\phi_i^{(T)} \tag{S55}$$

and

$$(\mathbf{b_{101}})_i = \mathbf{Q}_{101}\phi_i^{(T)} - a_{101}\mathbf{v}_T\phi_i^{(T)}\,. \tag{S56}$$

S1.3 Reduced equation

Since the system has a center manifold, we can express w as [1]

$$\mathbf{w} = \mathbf{h}(z, \mu) \approx \frac{1}{2}\mathbf{w_{20}} z^2 + \mathbf{w_{11}} \mu z + \frac{1}{2}\mathbf{w_{02}} \mu^2 \,, \tag{S57}$$

where $\mathbf{w_{20}}$, $\mathbf{w_{11}}$ and $\mathbf{w_{02}}$ are coefficients to be determined. To do so, we substitute the expression above into Eq. (S17). Specifically, for its left-hand side, we apply the chain rule $\frac{\mathrm{d}}{\mathrm{d}t}\mathbf{w} = \frac{\partial}{\partial z}\mathbf{h}\frac{\mathrm{d}}{\mathrm{d}t}z$, and substitute the expression given by Eq. (S16) for $\frac{\mathrm{d}}{\mathrm{d}t}z$. Then, we match the coefficients of z^2 and μz between the two sides of the equation, treating μ as a static variable. This yields the expressions

$$\mathbf{w_{20}} = -\mathcal{E}_0^{-1}\mathbf{b_{200}} \tag{S58}$$

and

$$\mathbf{w_{11}} = -\mathcal{E}_0^{-1}\mathbf{b_{101}} \,, \tag{S59}$$

where the invertibility of $\mathcal{E}_0$ is guaranteed by $\mathbf{b_{200}}$ and $\mathbf{b_{101}}$ lying entirely in the complementary subspace, since $\mathbf{q}^* \cdot \mathbf{b_{200}} = \mathbf{q}^* \cdot \mathbf{b_{101}} = 0$.

Finally, substituting Eq. (S57) into Eq. (S16) yields the reduced equation of the system on the center manifold:

$$\frac{\mathrm{d}}{\mathrm{d}t} z = C_{11}\mu z + C_{20} z^2 + C_{21}\mu z^2 + C_{30} z^3 \,, \tag{S60}$$

where we have put

$$C_{11} = a_{101} \tag{S61}$$

$$C_{20} = \frac{1}{2} a_{200} \tag{S62}$$

$$C_{21} = \frac{1}{2} a_{201} + \frac{1}{2}\left(\mathbf{a_{011}} \cdot \mathbf{w_{20}}\right) + \left(\mathbf{a_{110}} \cdot \mathbf{w_{11}}\right) \tag{S63}$$

$$C_{30} = \frac{1}{6} a_{300} + \frac{1}{2}\left(\mathbf{a_{110}} \cdot \mathbf{w_{20}}\right) \,. \tag{S64}$$

The cubic normal-form coefficient C_{30} explicitly depends on higher-order spectral moments of the critical Laplacian eigenmode. In particular, the direct cubic contribution contains the term $a_{300} = \mathbf{v}_T^* \mathbf{Q}_{300} S_4^{(T)}$, where $S_4^{(T)}$ denotes the fourth-order Laplacian moment. Since the sign of C_{30} determines whether the bifurcation is supercritical or subcritical, variations in $S_4^{(T)}$ can directly modify the bifurcation character through their contribution to a_{300}. This moment quantifies the localization strength of the critical mode and therefore provides a direct spectral link between network topology and the nonlinear onset of localized oscillatory states.

It is worth noting that the stable component w is not neglected in the reduction procedure. Since $\mathbf{w} = \mathbf{h}(z, \mu)$ is slaved to the center variable z through higher-order terms, its influence is implicitly incorporated into the reduced coefficients through $\mathbf{w}_{20}$ and $\mathbf{w}_{11}$. In particular, the cubic coefficient C_{30} consists of two structurally distinct contributions: the direct cubic projection term $\frac{1}{6}a_{300}$ and the indirect feedback term $\frac{1}{2}(\mathbf{a}_{110} \cdot \mathbf{w}_{20})$, the latter arising from the coupling between the center dynamics and the stable manifold correction.

An analysis of the reduced equation shows that, for $C_{11} > 0$, the equilibrium point $z = 0$ is stable when $\mu < 0$ and loses stability when $\mu > 0$. Also, if

$$C_{11}C_{30}\left(C_{11}C_{30} - C_{20}C_{21}\right) > 0 \tag{S65}$$

with

$$C_{30} < 0 \tag{S66}$$

$$C_{11} > 0 \tag{S67}$$

$$C_{20} < 0 \tag{S68}$$

$$C_{21} < 0 \,, \tag{S69}$$

then Eq. (S60) admits a positive equilibrium point $z = z_1 > 0$ that emerges from the bifurcation point and extends towards the homogeneous steady state $z = 0$ for $\mu < 0$, which corresponds to a supercritical bifurcation.

Conversely, if

$$C_{30} > 0 \tag{S70}$$
$$C_{11} > 0 \tag{S71}$$
$$C_{20} > 0 \tag{S72}$$
$$C_{21} > 0 \tag{S73}$$
$$C_{11}C_{30} > C_{20}C_{21}\ , \tag{S74}$$

then the positive branch extends toward $z = 0$ for $\mu > 0$, and the initial equilibrium is unstable, indicating a subcritical bifurcation.

S2 Numerical Methodology

S2.1 Numerical continuation

The overall continuation procedure is summarized as follows:

1. Generate the network Laplacian matrix in MATLAB.
2. Implement the $2N$-dimensional ODE system in AUTO-07P [2].
3. Initialize continuation from the homogeneous equilibrium.
4. Detect branch points (BP) and Hopf bifurcation points (HB).
5. Perform branch switching at the detected bifurcation points and continue the secondary solution branches.
6. Export representative solutions for subsequent visualization and direct numerical simulations.

The system dimension of the continuation problem was specified by

$$NDIM = 2N,$$

where N denotes the number of network nodes. The continuation step size was initialized with

$$DS = 10^{-4},$$

and adaptively controlled by

$$DSMIN = 10^{-8}, \qquad DSMAX = 10^{-3}.$$

This adaptive strategy improves computational efficiency while ensuring reliable detection of bifurcation points. The Newton correction tolerance was set to

$$EPSU = 10^{-7},$$

which was used as the convergence criterion for the continuation iterations.

During the continuation process, stability information is computed automatically by AUTO-07P. For steady-state branches, stability is determined from the eigenvalues of the Jacobian matrix, whereas for periodic branches it is determined from the corresponding Floquet multipliers.

S2.2 Numerical algorithm and convergence

Numerical simulations were performed in MATLAB using the explicit Forward Euler method. Initial conditions were taken from representative solutions obtained by numerical continuation, and the resulting $2N$-dimensional system of ordinary differential equations was integrated in time.

For the Forward Euler algorithm, numerical stability depends on the spectral radius $\rho(\mathbf{L})$ of the network Laplacian matrix L. Specifically, the time step must satisfy the CFL-type stability condition [3]

$$\Delta t < \frac{2}{D_{\max}\rho(\mathbf{L})}, \tag{S75}$$

where $D_{\max}$ denotes the maximal diffusion coefficient. Consequently, the admissible time step depends indirectly on the network size through $\rho(\mathbf{L})$, since the spectral radius generally increases as the network size becomes larger. Under this condition, the Forward Euler method is numerically stable and convergent.

For example, for networks with $N = 500$, 1000, and 5000 nodes, the corresponding stability thresholds are approximately 0.1797, 0.1217, and 0.0469, respectively. In all numerical simulations presented in this work, we consistently use a time step of $\Delta t = 0.01$, thereby satisfying the above stability criterion and ensuring numerical convergence.

Finally, for the purpose of reproducibility, we summarize the parameter settings used in all numerical simulations in Table S1. The table provides the specific parameter values adopted for each figure, including both the fixed model parameters and the continuation parameters.

Table S1: Summary of model parameters used in different simulations. The parameter values adopted in each figure are provided.

Parameters	Model	Figure
$\varepsilon = 0.055,\ \sigma = 2.4$ [4]	Brusselator	Fig. 1
$p = 2.15,\ \varepsilon = 0.055,\ \sigma = 2.4$	Brusselator	Fig. 2–Fig. 5
$p = 2.22,\ \sigma = 2.6$	Brusselator	Fig. 6
$a = 0.1,\ \varepsilon = 0.01,\ d_u = 0.1,\ d_v = 1.0$ [5]	FitzHugh–Nagumo	Fig. 7
$\varepsilon = 0.055,\ \sigma = 2.4,\ p = 2.15,\ r = 7.8$	Brusselator	Fig. S1
$p = 2.22,\ \sigma = 2.6$	Brusselator	Fig. S2

S3 Robustness with Respect to Network Size

To further investigate whether the observed localized oscillatory patterns depend on the network size, we additionally perform numerical simulations on BA networks with different numbers of nodes. Specifically, we consider networks with $N = 100$, $N = 500$, and $N = 5000$, while keeping all dynamical parameters and network generation rules identical to those used for the BA network shown in Fig. 5a. The parameter values are chosen as $\varepsilon = 0.055$, $\sigma = 2.4$, $p = 2.15$, and $r = 7.8$. In all cases, the BA networks are generated from an initial fully connected core of five nodes.

Fig. S1 presents the corresponding spatiotemporal evolution together with the associated network topologies for different network sizes. It can be observed that the localized oscillatory structures persist across all tested values of N. Although the detailed spatial distribution and localization positions vary slightly due to the randomness of the generated networks, the qualitative dynamical behavior remains unchanged. These results demonstrate that the emergence of localized oscillatory patterns is robust with respect to the network size.

S4 Robustness of the Mechanism under Different ε

To further assess the range of applicability of the proposed mechanism, we perform a sensitivity analysis with respect to the diffusion parameter ε, which plays a key role in the

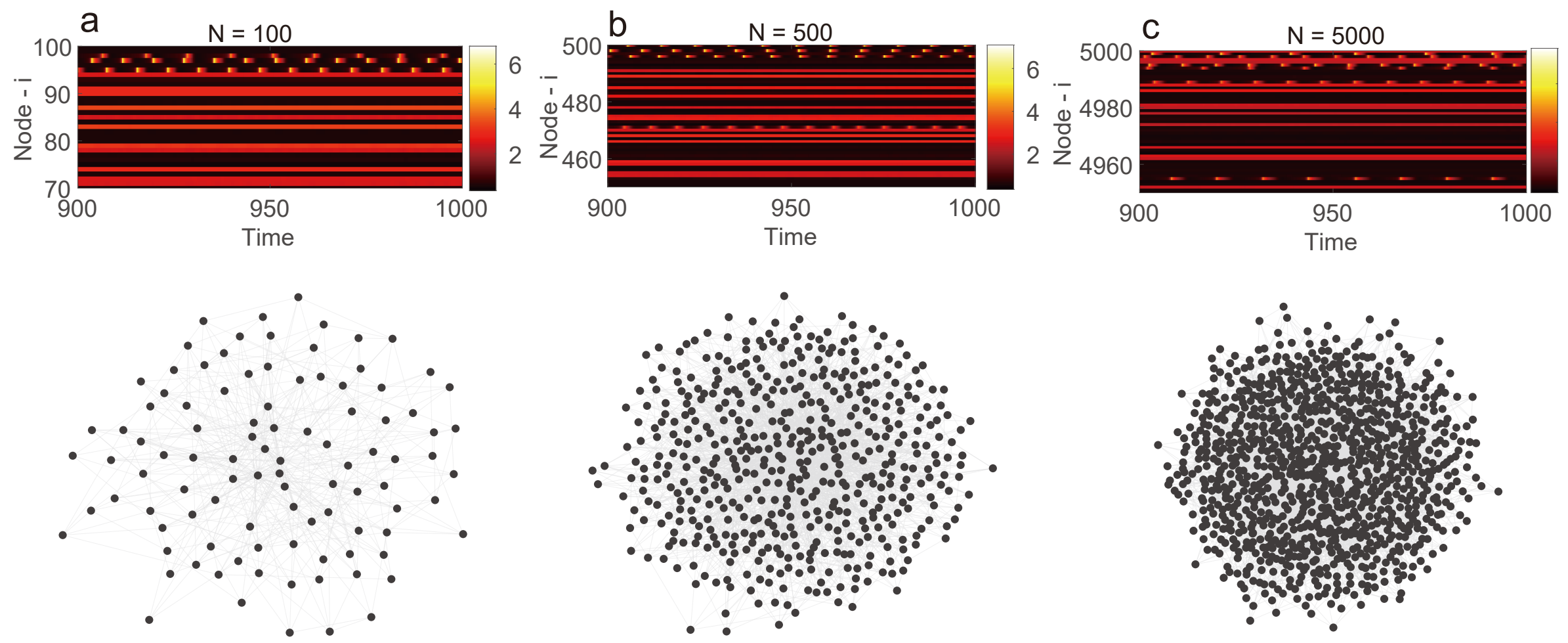

Figure S1: Robustness of localized oscillatory patterns with respect to the network size on BA networks. The upper panel in each subfigure shows the corresponding spatiotemporal pattern evolution, while the lower panel displays the associated network topology. The network sizes are **(a)** N=100, **(b)** N=500, and **(c)** N=5000, respectively. All simulations are performed using the same parameter set as that used for the $N = 1000$ BA network in Fig. 6a from the main text, namely, $\varepsilon = 0.055$, $\sigma = 2.4$, $p = 2.15$, and $r = 7.8$. The BA networks are generated using the same construction rule with an initial fully connected core of five nodes. The persistence of localized oscillatory structures across different network sizes demonstrates that the observed patterns are robust with respect to the network scale.

emergence of subcritical branches. Fig. S2 presents the resulting bifurcation structures and pattern evolution as ε varies, while all other parameters and the network topology are kept identical to those in Fig. 6.

As shown in Fig. S2a, no Turing instability is observed within the parameter regime of interest, and the dynamics are therefore governed solely by the Hopf mode, leading to spatially homogeneous temporal oscillations. With a slight increase in ε (Fig. S2b), a subcritical Turing branch begins to emerge. However, all steady patterned states remain unstable, so the system still evolves toward homogeneous oscillations. This indicates that stable Turing patterns are required for the emergence of localized spatiotemporal oscillations.

Through extensive numerical simulations, we identify an approximate critical value $\varepsilon \approx 0.024$ (Fig. S2c), marking the onset of stable localized patterns. In the ε range of Figs. S2c–d, secondary Hopf bifurcations on the subcritical branch are observed, giving rise to localized oscillatory patterns. For larger ε (Figs. S2e), the bifurcation disappears and such patterns are absent. This indicates that localized oscillations emerge only when the Hopf instability acts on the subcritical branch and induces the corresponding secondary Hopf bifurcation.

Furthermore, as ε increases (Figs. S2b–e), the snaking branch shifts toward larger r and the hysteresis loops progressively narrow. For sufficiently large ε (Fig. S2f), the hysteresis loop eventually disappears, and the system transitions to globally oscillatory dynamics rather than localized oscillations.

Overall, localized oscillations emerge only when Hopf instability coexist with stable localized patterns and give rise to a secondary Hopf bifurcation.

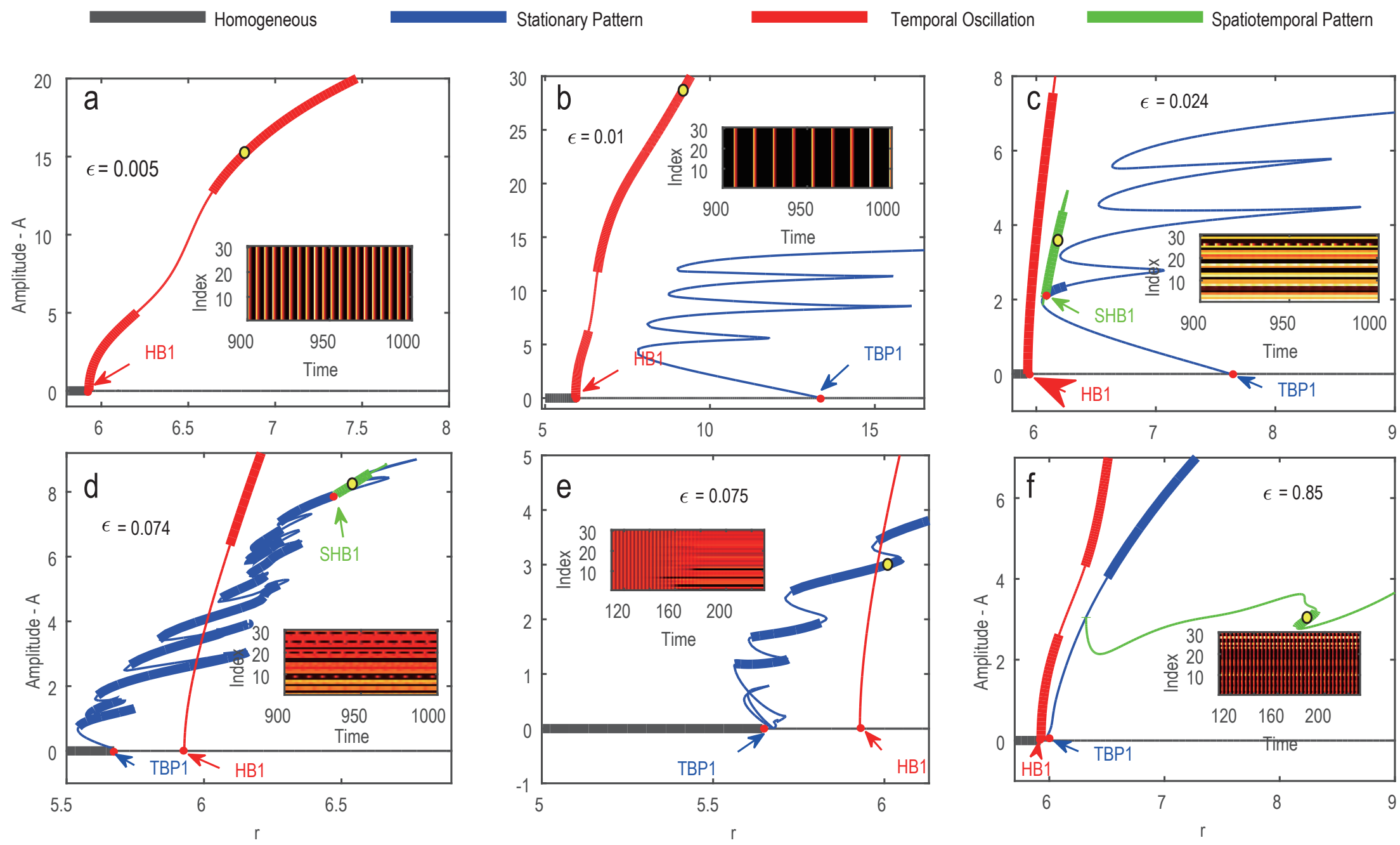

Figure S2: The applicable range of the parameter ε to the mechanism. Bifurcation structures and corresponding pattern dynamics for different ε, illustrating the robustness and parameter limits of the proposed mechanism. Panels **(a–f)** show the branch structures and the corresponding representative pattern evolution for increasing values of ε. Thick (thin) curves denote stable (unstable) solution branches. The black branch represents the spatially homogeneous steady state, on which the first Hopf bifurcation point (HB1) and the first Turing bifurcation point (TBP1) are indicated. The red branch corresponds to spatially homogeneous periodic oscillations emerging from HB1. The blue branch consists of steady Turing patterns bifurcating from TBP1, while the green branch represents spatiotemporal oscillatory patterns generated through Hopf bifurcation (SHB1) of the Turing branch. The spatiotemporal plots embedded in each panel show the numerical evolution of the system at parameter values marked by the yellow dots on the corresponding bifurcation diagrams. **(a)** For small ε, no Turing instability is detected in the considered parameter range, and the system evolves to spatially homogeneous oscillations. **(b)** Although a Turing bifurcation exists, all steady Turing states are unstable, and the dynamics still converge to spatially homogeneous oscillations. **(c)** At a critical value ($\varepsilon \approx 0.024$), a few stable localized Turing patterns emerge, and localized oscillatory states begin to appear. As ε increases further **(c–d)**, the number and parameter range of stable localized states increase, and localized oscillatory patterns are observed throughout this range. **(e)** the snaking structure and associated hysteresis loops gradually shrink, and no stable Hopf bifurcation is observed on the Turing branch; the unstable spatially homogeneous oscillations emerging from HB1 eventually evolve into steady spatial patterns. **(f)** For sufficiently large ε, both the snaking structure and localized states disappear, leading to globally oscillatory dynamics. Other parameters $p = 2.22$ and $\sigma = 2.6$.

References

[1] Kuznetsov, YA. *Elements of Applied Bifurcation Theory.* New York: Springer New York, 1995.

[2] Website. *AUTO-07P: Software for Continuation and Bifurcation Problems.* `https://github.com/auto-07p/auto-07p` (16 August 2026, date last accessed).

[3] Quarteroni A, Sacco R, Saleri F. *Numerical mathematics.* New York: Springer Science & Business Media, 2006.

[4] Kostet B, Tlidi M, Tabbert F, *et al.* Stationary localized structures and the effect of the delayed feedback in the Brusselator model. *Philos T R Soc A* 2018; 376: 1-18.

[5] Cebrián-Lacasa D, Parra-Rivas P, Ruiz-Reynés D, *et al.* Six decades of the FitzHugh-Nagumo model: A guide through its spatio-temporal dynamics and influence across disciplines. *Phys Rep* 2024; 1096: 1-39.

[6] Mimar S, Juane MM, Park J, *et al.* Turing patterns mediated by network topology in homogeneous active systems. *Phy Rev E*, 2019; 99: 062303.
